# In Vitro and In Vivo Effects of IGF-1 Delivery Strategies on Tendon Healing: A Review

**DOI:** 10.3390/ijms24032370

**Published:** 2023-01-25

**Authors:** Iris Miescher, Julia Rieber, Maurizio Calcagni, Johanna Buschmann

**Affiliations:** Division of Plastic Surgery and Hand Surgery, University Hospital Zurich, Sternwartstrasse 14, 8091 Zurich, Switzerland

**Keywords:** tenocytes, stem cells, growth factor, collagen, PI3K/Akt/mTOR pathway, Ras-MAPK pathway, PLC pathway

## Abstract

Tendon injuries suffer from a slow healing, often ending up in fibrovascular scar formation, leading to inferior mechanical properties and even re-rupture upon resumption of daily work or sports. Strategies including the application of growth factors have been under view for decades. Insulin-like growth factor-1 (IGF-1) is one of the used growth factors and has been applied to tenocyte in vitro cultures as well as in animal preclinical models and to human patients due to its anabolic and matrix stimulating effects. In this narrative review, we cover the current literature on IGF-1, its mechanism of action, in vitro cell cultures (tenocytes and mesenchymal stem cells), as well as in vivo experiments. We conclude from this overview that IGF-1 is a potent stimulus for improving tendon healing due to its inherent support of cell proliferation, DNA and matrix synthesis, particularly collagen I, which is the main component of tendon tissue. Nevertheless, more in vivo studies have to be performed in order to pave the way for an IGF-1 application in orthopedic clinics.

## 1. Introduction

Tendons connect muscles to bones and are able to transmit force, enabling motion. They consist of cross-linked collagen I (col I) fibres in a hierarchical manner, surrounded by an epitenon [1]. Upon damage, tendons are inclined to heal slowly due to low vascularization and low metabolic rate of the tendon cells [2]. Often, tendon healing is accompanied by two major problems, i.e., (i) adhesion formation, and (ii) scarring, resulting in mechanical inferior tissue—and eventually even in re-rupture [3].

The healing of tendons can be divided into three phases [1]. Briefly, there is a short first phase of inflammation, with increase in neutrophils and macrophages for 1–3 days. Second, a 6-week cellular and reparative phase follows, where tenocytes build up a collagen 3-dominated matrix in a non-organized way, accompanied by tendon swelling and high water and proteoglycan content. Only in the last phase (remodelling), cell density declines again and collagen III (col III) is replaced by collagen I. Fibre orientation gets more aligned in the direction of force transmission [4]. Notably, even if regenerative proper healing dominates, minimum period until full recovery is three months, and many reports mention half a year depending on the tendon under view [5] up to a full year until satisfactory outcome [6].

During tendon healing, growth factors and their dynamics play a non-negligible role [7]. After injury, platelets (thromobocytes) release different growth factors, such as bone morphogenetic proteins (BMPs), transforming growth factor betas (TGFβs), fibrobalst growth factors (FGFs), platelet-derived growth factors (PDGFs), vascular endothelial growth factor (VEGF), and insulin-like growth factor (IGF). Tendons undergo a well-orchestrated interplay of such growth factors during healing, and several signalling pathways are activated, with some overlapping phases and synergisms. While particularly BMP-12, BMP-13, and BMP-14, also denoted as growth and differentiation factors GDF-7, GDF-6, and GDF-5 [8], respectively, are crucial for growth and stem cell differentiation towards the tenogenic phenotype, BMP-2 is associated with fibrosis, very similar to some members of the TGF-β family, such as TGFβ2 and TGFβ3, and also connective tissue growth factor CTGF [9]. In contrast, TGFβ1 supports regenerative tendon healing and can activate IGF-1. Furthermore, PDGF-BB is highly important due to its mitogenic and chemotactic effects [10]; it attracts neutrophils to the wound site, accelerates proliferation and stimulates angiogenesis, a fundamental process especially during early tendon healing, before vessel ingrowth declines again in the final remodelling phase. Parallel to PDGF-BB, vascular endothelial growth factor VEGF is a key stimulus for (neo) angiogenesis [11].

In this narrative review article, we focus on IGF-1 and its mechanism of action in tendon healing. So far, IGF-1 is reported to have anabolic effects, to increase desoxy ribonucleic acid (DNA) and collagen synthesis; it is particularly important for adult tendon growth as a response to loading [12]. Since many decades, it has therefore been used to stimulate tenocytes in vitro [13] and to promote tendon healing in vivo [14]. In the first part, we address IGF-1 as a growth factor and summarize its main signalling pathways that are activated upon IGF-1 binding to its receptor. Then, we present in vitro studies that determined the effect of IGF-1 supplementation towards several cell types involved in tendon healing, such as tenocytes and mesenchymal stem cells (MSCs) [15,16]. Since the discovery of mesenchymal stem cells in tendons [17], their differentiation towards the tenocyte phenotype has gained high attention [18]. Hence, we compare the impact IGF-1 on tenocytes and MSCs (**PART A**). Finally, an overview over the most important pre-clinical and clinical in vivo studies is given (**PART B**), where among others also platelet-rich plasma (PRP) is addressed because it also contains IGF-1.

## 2. Methods

Consulting PubMed, the Web of Knowledge/Web of Science or the Google Scholar databases, roughly 90% (108 articles) of the papers in this review were found. The rest was found on other platforms, such as Embase, SCOPUS, or SportDiscus. Combinatorial key words were used, such as “tendon* AND IGF* OR insulin like growth factor”; “tenocyte* AND IGF * OR insulin like growth factor”; “IGF* AND stem cells*”; “IGF* AND multi-potency*”, during September 2021 to August 2022. Some papers (~20%) were cited in the references found within this search; others (~5%) were very recently published papers, which were included directly before submission if they were considered highly important for the topic. We focused on IGF-1 mechanism of action, including insulin-like growth factor binding proteins. Moreover, in vitro studies with IGF-1 or IGF-1 in combination with other growth factor application on tenocytes and stem cells, respectively, were addressed. Finally, in vivo application of IGF-1, but also of combinations with other growth factors, or based on platelet rich plasma (PRP) were discussed.

## 3. IGF-1 and Its Receptor IGF1R: Biological Characteristics and Mechanism of Action

IGF-1 is a hormone protein produced in the liver, when stimulated by growth hormone (GH) [19,20], and has a molecular weight of IGF-1 is 7649 Da. As its name implies, it has a similar structure like insulin [21] and binds not only to its own receptor IGF1R, but also to insulin receptor—and vice versa [21]. However, the binding affinities for IGF1R and insulin receptor, respectively, are different, with a high affinity of IGF-1 for IGF1R and an approximately 10 times lower affinity for insulin receptor [22]. On the other hand, the affinity of insulin for its own receptor is around 100 times higher compared to IGF1R [22]. Moreover, although similar in structure, IGF-1 and insulin exhibit different tissue distribution, different internalization kinetics and different subcellular distributions of hormone receptors [21]. Hence, both hormones can influence similar pathways, but to a different extent, and in addition activate also further pathways downstream differentially.

IGF-1 is responsible for cell growth and differentiation, as well as cell survival, protein synthesis, cell motility and proliferation. Three isoforms of IGF-1 exist: IGF-1Ea, IGF-1Eb, and IGF-1Ec, respectively [23], differing in the E domain of the peptide at the C terminus. IGF-1Ec is also called mechano-growth factor (MGF) because it is highly force-sensitive [24]. Besides this pronounced mechano-sensitivity, it also acts as an important regenerative factor by activating stem cells [25,26].

Concerning IGF-1 association, IGF binding proteins (IGFBPs) are important because they regulate the IGF-1 activity by keeping circulating IGF-1 concentrations in check [27]. Seven binding proteins are known, denoted as IGFBP1–IGFBP7, where the GH-dependent IGFBP3 binds IGF-1 most strongly, with approximately 80% of IGF-1 bound to IGFBP3 [28]. As for IGFBP4, it is increased in long distance runners as an effect of mechanical loading and occurs co-localized with IGF-1 in tendons [29]. Moreover, during tendon injury, IGFBPs 2–4 are increased, particularly one month after laceration [30]. Compared to binding affinity for its receptor IGF1R, IGF-1 binds to IGFBP2 [31] and IGFBP 5 [32] with a higher affinity, so that increases in those two binding proteins decrease IGF-1 activity [33].

**Figure 1:**
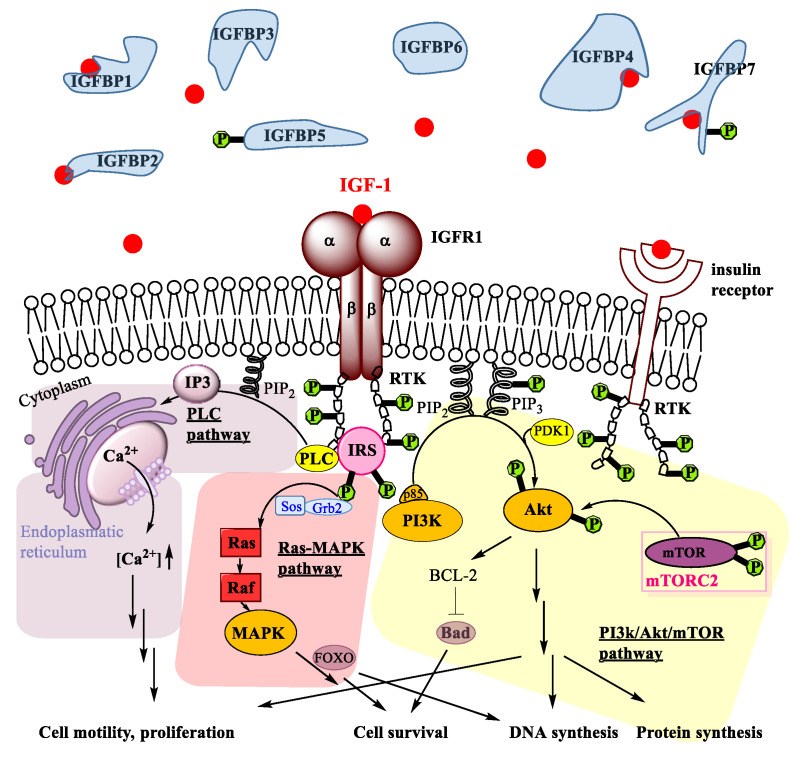
Signalling pathways activated through IGF-1 binding to its receptor IGFR1. PI3K/Akt/mTOR pathway (shaded yellow); Ras-MAPK pathway (shaded red); and PLC pathway (shaded violet). Further explanations are given in the main text.

Regarding the mechanisms of action, IGF-1 binding to IGF1R receptor regulates several pathways. The IGF1R receptor is a hetero-tetramer with two extracellular α subunits and two transmembrane β subunits. Upon binding of IGF-1, the receptor tyrosine kinases (RTKs) are activated [34]. The tyrosine residues of RTKs are bound to the β subunits of IGFR1 and are phosphorylated upon IGF-1 binding to IGF1R (Figure 1; see Abbreviations at the end of the manuscript). Like this, such phosphorylated tyrosine binding domains can be recognized by different docking proteins, such as insulin receptor substrates (IRS) [35], which themselves also get phosphorylated.

Besides several crosstalks [22] of non-negligible importance for some viral effects in oncogenic progression [36], three pathways are activated upon IGF-1 binding to IGF1R: the PI3 kinase/Akt pathway [37], the Ras-MAPK pathway [38] and the PLC pathway [39]. As for the phosphatidylinositol 3-kinase (PI3K) pathway, the IRS interact with p85, which is a regulatory subunit of PI3K. This induces the phosphorylation of phosphatidylinositol diphosphate (PIP_2_) to phosphatidylinositol triphosphate (PIP_3_) (Figure 1). Subsequently, PIP3 activates PDK1, which in turn phosphorylates a tyrosine of survival protein kinase B (Akt kinase). It has to be emphasized, however, that full activation of Akt is only achieved by a second phosphorylation induced by mammalian Target of Rapamycin Complex 2 (mTORC2), which regulates proliferation and cell survival, migration and cytoskeletal remodelling [40]. Upon activation of Akt, several downstream substrates are activated, which finally lead to DNA synthesis, protein synthesis, cell survival, and differentiation. Notably, phosphorylation and activation of B-cell lymphoma-2 BCL-2 antagonist of cell death BAD [41] by Akt mediates the IGF-1 induced cell survival activity. Hence, the PI3K/Akt/mTOR pathway is a prominent pro-survival pathway. It has been reported that this pathway is activated in tendons upon IGF-1 binding and that the signalling is essential for proper adult tendon growth because IGF-1 regulates tenocyte proliferation and collagen synthesis via PI3K/Akt/mTOR [12] and indirectly via TGFβ1 [42].

Parallel to the PI3K/Akt/mTOR pathway, IGF-1 binding to IGF1R also activates the Ras-MAPK pathway (Figure 1). Here, IRS interact with adapter protein Grb2 and associated Sos guanine nucleotide [43] to activate the small GTPase Ras [44], which then activates Raf [45], a key mediator of response to growth factors in general [46], such as epidermal growth factor EGF, fibroblast growth factor FGF or platelet-derived growth factor PDGF, respectively. In several steps, mitogen-activated protein kinase (MAPK) is then activated. In tenocytes, particularly cell survival and proliferation has been attributed to FOXO activation downstream MAPK [47,48,49]. Activation of MAPK overall leads to increased DNA synthesis and cell survival, very similar to the PI3K/Akt/mTOR pathway.

Furthermore, IGF-1 can also act via the phospholipase C (PLC) pathway. RTKs activate the PLC, upon which PLC hydrolyses PIP_2_ to result in inositol trisphosphate (IP3) that binds to the Ca^2+^ channel IP3 receptor [39]. Like this, Ca^2+^ is released from the storage in the endoplasmatic reticulum (Figure 1). Overall, activation of the PLC pathway by IGF-1 leads to a calcium ion influx into the cytosol [50]. Due to the close proximity of the endoplasmatic reticulum and the mitochondria, localized high concentration of Ca^2+^ facilitates uptake into the mitochondria, where this leads to conformational changes and to cell motility [51], supporting cell migration and cell proliferation.

The overall influence of IGF-1 on cell metabolism—in coordination with GH and insulin—can be summed up by a variety of bioactivities that increase anabolism. Nutritionally regulated like insulin [40], IGF-1 is involved in the glucose homeostasis [52,53]. It has been reported to reduce blood glucose levels [54], and infusion of rhIGF-1 to patients has been associated with increased glucose utilization as well as reduced insulin secretion, resulting in an increased insulin sensitivity [55]. Furthermore, it participates in protein metabolism [53] and lipolysis, particularly increasing synergistically the effect of GH on lipolysis and ketogenesis [56]. Finally, consistent evidence exists for the inverse relationship of IGF-1 levels and metabolic syndrome, with very low IGF-1 accompanied to metabolic syndrome [57,58].

Besides the described anabolic effects induced by IGF-1, it has to be noted that IGF-1 bioactivity has been determined also to decrease catabolism. IGF-1 is involved in the inhibition of extracellular matrix degradation, where it has been found to block multiple degradative effects. IGF-1 has been reported to reduce GAG release from ECM by around 50% [59] and to furthermore block collagen release from ECM, while downregulating matrix metalloproteases MMP-1, MMP-3, MMP-8, and MMP-13, respectively [60]. Another study has reported an inhibition of the loss of sulfated GAGs and of collagen under IGF-1 administration [61]. Finally, cell apoptosis induced by collagen release from ECM has been shown to be inhibited under IGF-1 [62].

## 4. PART A: Effects of IGF-1 Delivery In Vitro

In the following part A of this review article, we have screened literature on the IGF-1 effects on several cell types that are involved in tendon healing in vitro, i.e., tenocytes, synoviocytes, and MSCs. In addition, combinatorial application of several growth factors, including IGF-1, were discussed. It will be shown that time point, concentration and single versus repetitive stimulation may be important when the impacts of IGF-1 are determined.

In vitro studies have shown that growth factors such as IGF-1 play an important role in tendon healing, tendon regeneration, and in the normal development of tendon tissue [63,64,65,66]. IGF-1 is produced in several tissues, also in tendon [67] where its expression increases in response to injury, vibration trauma, and mechanical load [68,69,70]. In addition to its role as potent mitogen, IGF-1 has been shown to increase extracellular matrix formation [66,69,71] and to act as chemoattractant on tenocytes [72] and on MSCs [73,74,75]. Due to these positive effects, IGF-1 is often used for in vitro experiments, either individually or in combination with other growth factors.

### 4.1. IGF-1 Supplemented Culture Media

#### 4.1.1. Proliferation and Migration

##### Stem Cells

**Figure 2:**
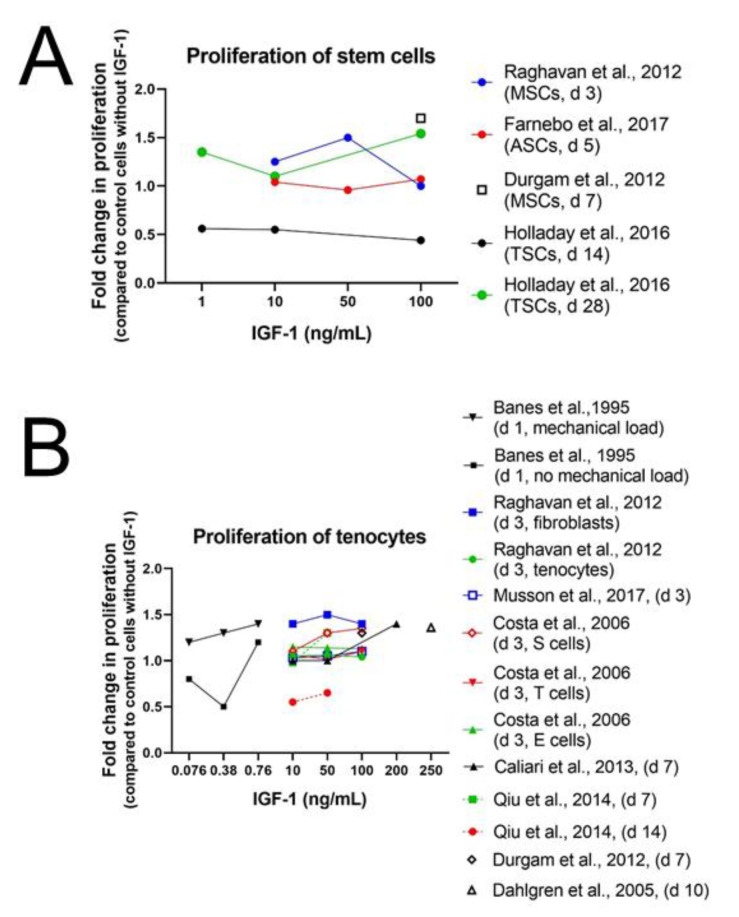
Human ASCs in Ham’s F12 containing 0.2% bovine serum albumin BSA [77]; *rat* ASCs in ECM-solution containing 10% FCS [79]; equine MSCs in high-glucose DMEM containing 10% FBS and AA (37.5 µg/mL) [76] and *rat* TSCs in basal TSC medium [78] (**A**). Avian tenocytes in DMEM without FCS [13]; human tenocytes in Ham’s F12 containing 0.2% BSA [77]; *rat* tenocytes in DMEM/F12 containing 0.5% FBS [80]; rabbit tenocytes in Hams’s F12 containing 0.2% BSA [81]; equine tenocytes in DMEM without FCS [82]; human tenocytes in α-MEM without FBS [83]; equine tenocytes in high-glucose DMEM containing 10% FBS and ascorbic acid (AA) (37.5 µg/mL) [76]; M-199 medium containing 5% FBS and AA (100 µg/mL) [84] (**B**). For better view, see also SI Figure S1.

Results from Durgam et al. [76] and Raghavan et al. [77] confirm the positive effect of IGF-1 on cell proliferation. Equine MSCs, cultured with 100 ng/mL IGF-1 on a tendon matrix for 7 days, showed higher cell proliferation than cells without IGF-1 supplementation and also human adipose-derived stem cells (ASCs) proliferated more upon addition of IGF-1 (10, 50, and 100 ng/mL) than ASCs in the control medium on day 3 (Figure 2A). This effect was not dose-dependent though and was highest with 50 ng/mL compared to 10 and 100 ng/mL (Table S1). In contrast to the human stem cells, *rat* patellar tendon-derived stem cells (TSCs) did not show increased cell proliferation upon IGF-1 exposure (1, 10 and 100 ng/mL). Proliferation from day 0 to 14 was significantly less in the 100 ng/mL group than in the untreated cells. Fold change in cell numbers from seeding to analysis with 1 or 10 ng/mL IGF-1, respectively at day 14 and independently from the concentration at day 28 remained unaffected [78]. However, another experimental setup with *rat* ASCs confirmed the positive results of IGF-1 obtained with human ASCs. Lentivirus transduced *rat* ASCs reseeded in a tendon-specific hydrogel from human cadaver tendons had a significantly larger cell proliferation with best results using 100 ng/mL IGF-1 compared to cells without IGF-1 supplementation in serum-free media. However, optical density (OD) values of MTT proliferation assays with 100 ng/mL were not significantly higher than with 10 ng/mL and reached approximately the same intensity as for cells cultured in medium with fetal calf serum (FCS) without addition of IGF-1 [79]. Over all, IGF-1 seems to be able to improve cell proliferation of stem cells at higher concentrations but not all types of stem cells react in the same way. For instance, *rat* TSCs remained unaffected by treatment with 1 or 10 ng/mL IGF-1 and showed even a reduced cell proliferation with 100 ng/mL IGF-1.

##### Tenocytes and Fibroblasts

Proliferative effect of IGF-1 has not only been detected in stem cells but also in differentiated tendon derived cells (Figure 2B and SI Figure S1). Equine tenocytes with 100 ng/mL IGF-1 as well as human tenocytes and fibroblasts enhanced proliferation upon IGF-1 treatment [76,77] (Table S2). Raghavan et al. could show higher cell proliferation for human tenocytes and fibroblasts on day 3 of culture in Ham’s F12 media containing 0.2% BSA. Like for human ASCs, cell proliferation was not dose-dependent either and highest values were as well reached with 50 ng/mL. The importance of sera in culture media on the effect of IGF-1 supplementation demonstrate the experiments of Qiu et al. [84]: culture of human tenocytes in serum-free media supplemented with IGF-1 (10 or 50 ng/mL) was able to maintain surviving tenocyte numbers but did not increase cell proliferation. Although cell number in serum-free culture containing IGF-1 was significantly higher after 14 days compared with cells in serum-free media without IGF-1, where no viable tenocytes were detectable, cell number increase was not comparable to that observed in the positive control cultures with 10% FBS. In addition, tenocytes from other species, such as rabbit tenocytes, showed enhanced proliferation with IGF-1 supplementation (10, 50, and 100 ng/mL). Interestingly, slight cell type-dependent effects could be detected. Cells from the synovial sheath displayed a positive dose–response curve to IGF-1 but not to basic fibroblast growth factor (bFGF). Conversely, cells from the epitenon did not display a positive dose–response to IGF-1 but to bFGF and cells from the endotenon did not display a positive dose–response either to bFGF nor to IGF-1, demonstrating the high specificity of growth factors [81,85] (Figure 2B). A shorter time frame was used for experiments with *mouse* tenocytes using 100 ng/mL IGF-1. While after 1 h no change in proliferation could be detected, expression of proliferation Marker Ki67 (Mki67) was 7 times increased at the latest measurement after 24 h, confirming the results in human and rabbit tenocytes [12]. In contrast to human and rabbit tenocytes, *rat* tenocytes treated with IGF-1 (10, 50, 100 ng/mL) cultured in medium containing 0.5% fetal bovine serum (FBS) did not proliferate more than tenocytes without IGF-1 supplementation [80].

To enhance scaffold strategy, soluble factor supplementation is widely used. A positive dose-dependent proliferation effect and increased metabolic activity upon IGF-1 treatment (10, 50, and 200 ng/mL) was observed on equine tenocytes seeded on collagen-glycosaminoglycan (CG) scaffolds after 7 days. Additionally, IGF-1 induced significant increase in tenocyte migration into anisotropic CG scaffold compared to the non-supplemented, serum-free media control for all dose levels [82]. Experiments on equine explant cultures confirm the positive effect of IGF-1 on cell proliferation in horse tenocytes as treatment with 250 mg/mL IGF-1 resulted in an increased DNA content [86].

Studies on tenocytes of genetically modified mice confirm the anabolic role of IGF-1 in tendon. Disser et al. could demonstrate the anabolic role of IGF-1 in tendon also in genetically modified mice in which IGF1R was conditionally deleted in SCX-expressing tenocytes using a tamoxifen-inducible Cre-recombinase system [12]. Achilles tendon was removed from the animal, resulting in compensatory hypertrophy of the synergist plantaris muscle and tendon (surgical overload) and in consequence, a neotendon area of new tendon matrix was formed around the original tendon. The total tendon cross-sectional area (CSA) was not different between groups after one week, but two weeks later the total CSA was twice as large in mice expressing IGF-1 receptor (SCX:IGF1R^+^) as in mice lacking the IGF-1 receptor (SCX:IGF1R^Δ^). This change occurred due to a greater expansion of the neotendon over time in Scx:IGF1R^+^ mice, whereas SCX:IGF1R^Δ^ mice displayed no change between day 7 and day 14. Additionally, the percentage of proliferating cells was 2-fold greater in the neotendon of Scx:IGF1R^+^ mice compared with Scx:IGF1R^Δ^ mice, whereas cell density was not affected in the original tendon, neotendon, or total tendon across time or genotype [12].

Like surgical overload also mechanical loading influences healing and regeneration of tendon tissue. A study on avian cells from the epitenon and internal compartment (TSCs) and tendon internal fibroblasts (TIF) of flexor tendons shows synergistic effects of cyclic mechanical loading in combination with IGF-1 supplementation. IGF-1 (10, 50, or 100 pM = 0.076, 0.38, 0.76 ng/mL) was only a modest mitogen for TSCs but mechanical loading increased DNA synthesis additionally at any IGF-1 concentration. The same effect even more prominent was observed for TIFs at doses below 100 pM. Cell proliferation was highest in the 100 pM treated group, however, no difference in DNA synthesis could be detected between the 100 pM treated group with mechanical loading compared to group with the same treatment but without mechanical loading [13]. In summary, tenocytes and tendon fibroblasts of many species, such as human, rabbit, mice and horse, show increased cell proliferation upon IGF-1 supplementation. Nevertheless, there exist some differences among species, as in *rat* and *avian* tenocytes addition of IGF-1 did not lead to a higher cell proliferation. Interestingly, combination of IGF-1 with mechanical load had a synergistic effect and was able to increase cell proliferation in avian tenocytes. These results demonstrate that not only the origin of cells but also experimental settings influence the proliferative effect of IGF-1.

#### 4.1.2. Differentiation and Tendon Markers

##### Stem Cells

**Figure 3:**
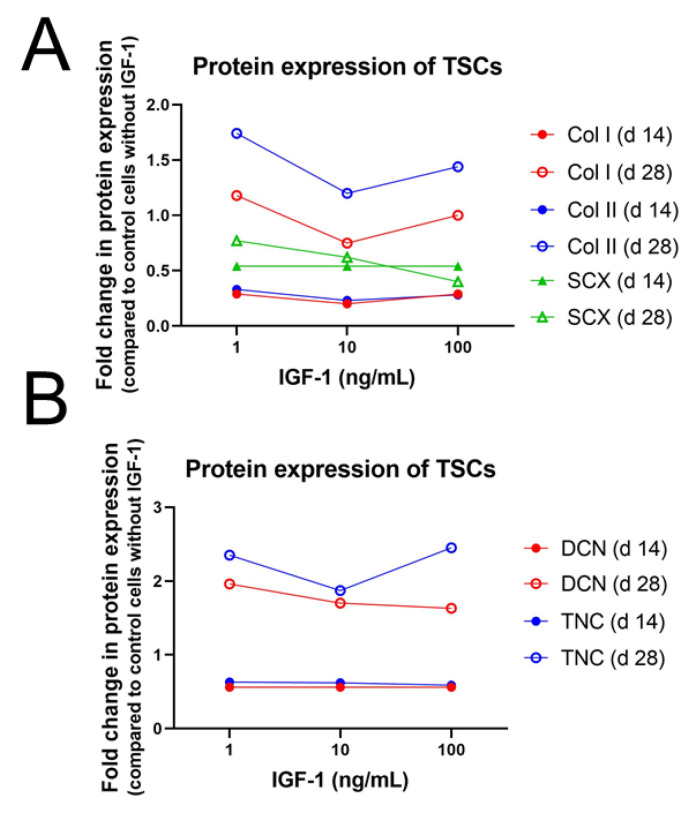
Impact of IGF-1 concentration on the protein expression of collagen I and II (col I and col II) and Scleraxis (SCX) (**A**) as well as Decorin (DCN) and Tenascin-C (TNC) after 14 or 28 days in *rat* tendon stem cell culture (TSC) in basal TSC medium [78] (**B**).

Differentiation of stem cells into a tissue-specific lineage is often used in cell therapies and tissue engineering. Progress has been made in osteogenic and chondrogenic differentiation but induction of stem cells into a tenogenic fate remains challenging. IGF-1 treatment of *rat* ASCs could maintain multipotency up to 28 days and expression levels of some, but not of all, tendon-, bone-, and cartilage-related proteins [78]. There was no significant loss in adipogenic and osteogenic potential after treatment with 10 ng/mL IGF-1 for 14 and 28 days and osteogenic potential was maintained with all doses of IGF-1 (1, 10, and 100 ng/mL) after 28 days. The amount of CD90 positive cells, an MSC surface marker, was not affected by IGF-1 supplementation, except for a transient decrease in the fraction of treated cells with 100 ng/mL on day 10. In addition, there was no significant change of CD31 positive cells, a marker for endothelial cells present mainly in blood vessels and therefore a sign of increased vascularization and blood circulation. The amount of CD31 positive cells was significantly increased in the fractions of untreated cells after 14 and 28 days, while in the groups treated with 10 or 100 ng/mL IGF-1 no increase has been detected in comparison to the day 0 cells. Protein level of SCX increased up to day 28 for all doses of IGF-1 but values decreased with increasing IGF-1 concentration and were highest in the untreated group (Figure 3). In contrast, col I synthesis decreased significantly at all doses after 14 and 28 days except for day 28 with 1 ng/mL, when reduction was not significant. Correspondingly, col II production was significantly reduced at both time points with 10 ng/mL IGF-1 but protein levels of DCN, TNC, and osteonectin remained unaffected at any dose.

**Figure 4:**
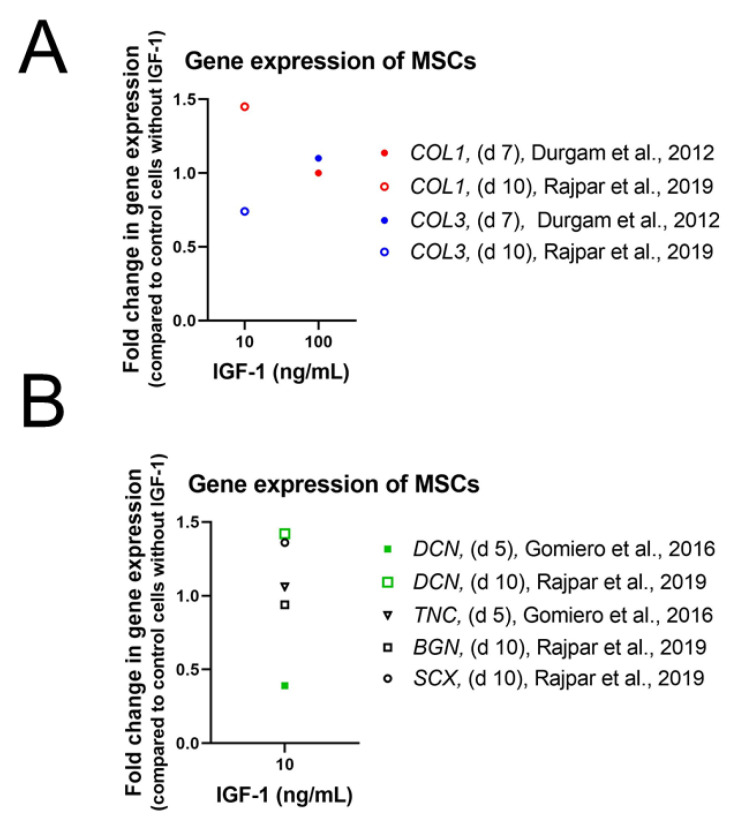
Impact of IGF-1 concentration on the gene expression of collagen I and III (*COL1* and *COL1*) (**A**), Decorin (*DCN*), Tenascin-C (*TNC*), Biglycan (*BGN*) as well as Scleraxis (*SCX*) (**B**) after 5, 7, or 14 days in equine mesenchymal stem cell culture in high-glucose DMEM containing 10% FBS and AA (37.5 µg/mL) [76]; equine MSCs in high-glucose DMEM containing 10% FBS and AA (37.5 µg/mL) [88]; and equine tenocytes in DMEM containing 20% FCS [87].

The limited potential of single IGF-1 supplementation to drive stem cells toward a tenogenic fate was confirmed as well in other species. In equine MSCs, gene expression levels of *COL1*, *COL3*, and *COMP* treated with 100 ng/mL IGF-1 remained unaffected [76] and also treatment with 10 ng/mL IGF-1 did not influence gene expression of tenogenic induction markers such as *DNC*, *TNC*, and *EGR1* [87]. Experiments with equine MSCs, seeded on a collagen gel and treated with 10 ng/mL IGF-1, confirm these observations as gene expression levels of *SCX*, *DCN*, *BGN*, *COL1*, and *COL3* did not differ from control cells [88] (Figure 4). Furthermore, Western Blot analysis of three-dimensional high-density cultures of canine MSCs did not show any synthesis of col I, col III, DCN, TNMD and SCX in contrast to canine tenocytes which served as control [89].

So far, often-used tenogenic culture medium consists of connective tissue growth factor (CTGF), TGFβ3 (transforming growth factor beta 3), BMP-12 (bone morphogenic protein 12), and AA (ascorbic acid) [90]. Obviously, no IGF-1 is used for standard tenogenic induction.

##### Tenocytes and Fibroblasts

**Figure 5:**
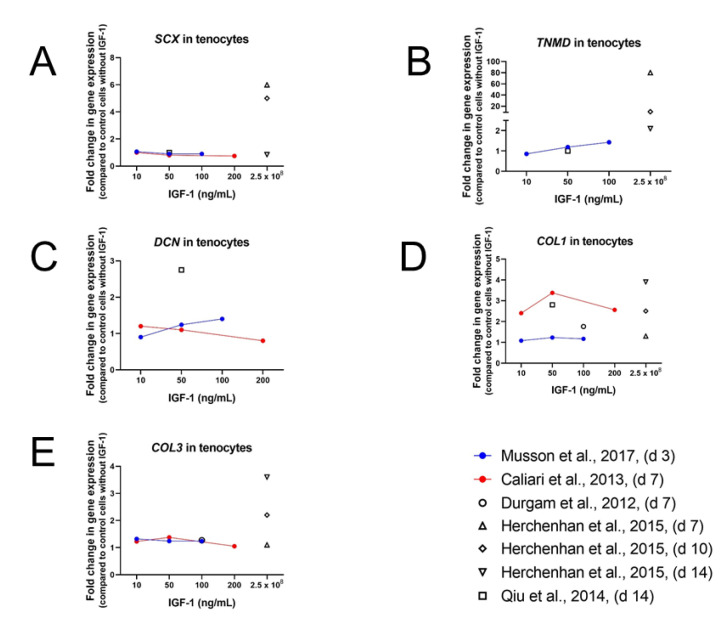
Impact of IGF-1 concentration on the gene expression of Scleraxis (*SCX*) (**A**), Tenomodulin (*TNMD*) (**B**), Decorin (*DCN*) (**C**), *COL1* (**D**), and *COL3* (**E**) after 3, 7, 10, or 14 days. Further information: *Rat* tenocytes in DMEM/F12 containing 0.5% FBS [80]; Equine tenocytes in DMEM without FCS [82]; equine tenocytes in high-glucose DMEM containing 10% FBS and AA (37.5 µg/mL) [76]; human tenocytes in DMEM/F12 containing 0.5% FBS [91]; and human tenocytes in α-MEM without FBS [84].

Studies using IGF-1 often differ in concentrations, time points of treatments and analysis, assessments, and species influencing the outcome of the experiments. In human tenocytes, cultured without FBS but with 50 ng/mL IGF-1, no differences in mRNA level of *SCX* and *TNMD* could be detected after 14 days (Figure 5A,B). On the other hand, *DCN* and *COL1* were upregulated after IGF-1 supplementation [84] (Figure 5). However, mRNA levels of *SCX* and *TNMD* in human tendon fibroblasts, cultured in coated plates with SYLGARD, a silicone elastomer, and in medium containing 0.5% FBS, were increased after 10 and 14 days of treatment with 250 mg/mL IGF-1 [91]. As in human tenocytes, in human fibroblasts *COL1* and additionally tested *COL3* mRNA levels were higher than levels in control cultures without IGF-1 on day 10 and on day 14. In addition to IGF-1, also the concentration of FBS in the culture medium influenced the gene expression levels in human tendon fibroblasts as experiments with long IGF-1 incubation (21 and 28 days) showed. A decrease of expression levels of *SCX*, *TNMD*, *COL1*, and *COL3* could be observed in medium containing 10% FBS after IGF-1 supplementation for 21 and 28 days, respectively. The same IGF-1 treatment with 0.5% FBS plus IGF-1 led to an increase of gene expression of *TNMD*, *COL1*, and *COL3* on d 21 and d 28. Expression levels of *SCX* though, were slightly downregulated after 21 days and only weakly upregulated after 28 days in media containing 0.5% FBS.

Results from Musson et al. [80] confirm the positive influence of IGF-1 on TNMD gene expression as *rat* tenocytes treated with 10, 50, or 100 ng/mL IGF-1 and 0.5% supplemented FBS showed increased TNMD levels on day 3. However, other tenocyte-related gene expression (*SCX*, *COL1*, *COL3*, *DCN*, and runt-related transcription factor-2 (*RUNX2*)) on day 3 remained unaffected after IGF-1 treatment at any dose. The expression of the osteoblastic marker alkaline phosphatase (*ALP*) and the chondrogenic marker aggrecan (*ACAN*) were significantly increased in a dose-dependent manner. On the other hand, expression levels of SOX9, a transcription factor essential for skeletal development among other things, decreased in a dose-dependent manner upon IGF-1 supplementation.

Consistently with the results of human tenocytes cultured in serum-free medium also equine tenocytes cultured in serum-free medium and supplemented with IGF-1 (10, 50, and 200 ng/mL) showed significantly upregulated levels of *COL1* [82]. The same effect could also be observed for *COL3*—but smaller than for *COL1* (Figure 5D,E). Measurements were carried out after 7 days and showed peak levels at the medium dose, which was also used in the experimental setting with the human tenocytes on day 14. In contrast to the significant upregulation of *DCN* in human tenocytes, mRNA level of *DCN* in equine tenocytes was only slightly upregulated at low dose and decreased dose-dependently at medium and high dose. The same result was obtained for Tenascin-C (TNC). Expression levels for *SCX* were not affected in human tenocytes with 50 ng/mL, which was also true for equine tenocytes with the lowest IGF-1 dose (10 ng/mL) on day 7. Higher IGF-1 concentration on the other hand, led to a dose-dependent downregulation of *SCX* levels in equine tenocytes, observed as well for cartilage oligomeric matrix protein (*COMP*). How important experimental settings for the outcome of the results are demonstrates the study of Durgam et al. carried out in equine MSCs as well [76].

In contrast to the results of Caliari et al., Durgam et al. did not find any difference in gene expression levels of *COL1*, *COL3*, and *COMP* [76]. However, experiments were carried out with 100 ng/mL IGF-1 instead of 10 ng/mL, culture media contained 10% FBS and mRNA was analyzed on day 10 instead of day 7. Early effect of 100 ng/mL IGF-1 on *mouse* tenocytes was analyzed after 1, 2, 6, and 24 h by Disser et al. [12]. An early upregulation of *SCX* (1 h and 2 h) could be observed, but after 24 h expression level did not differ from non-treated control group at 0 h. *COL1* and *COL3* were not affected at early time points, but—while expression levels of *COL1* remained stable—*COL3* values increased by 24 h. In contrast, expression levels of *COMP* did not differ in the beginning, but were lower at later time points (6 h and 24 h), whereas MKX was downregulated at all time points. *TNMD* levels were not affected and *BGN* and *TNC* showed only very little changes in expression patterns. All these results taken together, a positive effect on gene expression of tendon related genes, such as *COL1* and *COL3*, *DCN*, *TNMD*, *SCX*, and *TNC*, is often observed, though results are not always consistent, depending on duration of the experiment, time point of measurement, IGF-1 concentration, origin of cells, and amount of sera in the culture media, whereas lower FBS concentration goes ahead with higher gene expression levels.

#### 4.1.3. Morphology and ECM

##### Stem Cells

Morphological evaluations confirm the limited potential of IGF-1 supplementation without addition of other growth factors to drive stem cells into a tendon fate. While in 3D high-density cultures of canine tenocytes close cell-to-cell contacts were observed, MSC cultures treated with IGF-1 (10 ng/mL) showed little signs of intercellular contacts. In contrast to tenocytes, the intercellular space was filled only to some extent with unstructured extracellular matrix components. In comparison with untreated MSCs though, MSCs with IGF-1 supplementation did not exhibit morphological signs of apoptosis such as membrane-bebbling, free cellular organelles and other cellular debris [89].

Rat derived TSCs treated with 1, 10, or 100 ng/mL IGF-1 were capable of forming col II-expressing pellets after 28 days with a diameter of approximately 450 µm. However, protein analysis showed a decreased col II expression with 10 and 100 ng/mL IGF-1 supplementation at 14 and 28 days, but only with 10 ng/mL reduction was significant. Col I expression decreased as well at any dose, although this effect was only statistically significant after 28 days in the 10 and 100 ng/mL groups [78]. In contrast to *rat* TSCs, equine MSCs did not reduce collagen synthesis. Collagen amount remained unaffected and GAG content was even enhanced upon treatment with 100 ng/mL IGF-1 after 7 days [76]. Consistently, in equine MSCs treated with 10 ng/mL for 10 days gene expression level of col I and col III did not change but in contrast to the study from Durgam et al., GAG content was not enhanced [88]. In summary, IGF-1 did not adapt cell morphology to typical tenocyte appearance and collagen content remained either unaffected or was even reduced as could be shown in *rat* TSCs (Figure 3).

##### Tenocytes and Fibroblasts

In contrast to stem cells, where IGF-1 supplementation showed a negative effect on collagen content, addition of IGF-1 influenced ECM formation in tenocytes and fibroblasts predominantly positively, confirming the ability of IGF-1 to stimulate matrix production as it has been reported in several studies [65,66,71].

In human tendon fibroblasts cultured on plates coated with the silicone elastomer SYLGARD maturation of the extracellular matrix has been reported in the following groups from day 21 to day 28: (1) 0.5% FBS; (2) 0.5% FBS plus 250 ng/mL IGF-1; (3) 10% FBS and (4) 10% FBS plus 250 ng/mL IGF-1) [91]. Samples with the low FBS treatment and supplementation with IGF-1 showed a significantly higher collagen content compared to both 0.5% and 10% FBS-treated constructs. At 28 days, the group with 10% FBS plus IGF-1 also showed a significantly higher collagen content than the groups without IGF-1. In both IGF-1 supplemented groups, collagen content was higher at 28 days compared to 21 days. As for the collagen content, all treatments showed an increase in fibril diameter from days 21 to 28. At 21 days, IGF-1 supplementation affected fibril diameter positively compared to the FBS groups but overall, the patterns of content and diameter were similar with the highest value for 0.5% FBS+ IGF-1-treated samples. At 28 days, IGF-1 supplemented constructs showed significantly higher collagen content but reduced fibril diameter compared to only FBS-treated samples. Looking at the early phase from day 7 to day 14, the total collagen content developed from very low values at 7 days to higher values at day 14. Thereby, the 0.5% FBS plus IGF-1 treatment group showed increased values at 10 and 14 days compared to the 0.5% FBS groups, with a significant effect of the treatment over time. In another experiment using human tenocytes though, the positive effect of IGF-1 on ECM formation could not be detected [84]. In comparison to the positive control group cultured in media containing 10% FBS, tenocytes in serum-free media supplemented with 10 or 50 ng/mL IGF-1 for 14 days exhibited less aggregated collagen layers and the Sirius red staining was clearly weaker than in the positive controls, what was confirmed by quantification. However, tenocytes in serum-free media containing IGF-1 independent from its concentration exhibited elongated, spindle-shaped, fibroblast-like appearances and did not show round shape, which is indicative of a phenotypic drift of tenocytes as described by Yao et al. [92].

On the other hand, the ability of IGF-1 to stimulate matrix production could be confirmed in horse explant cultures treated with either 250 ng/mL IGF-1, 3 mg BAPN/mL (beta-aminopropionitrile, a lysyl oxidase inhibitor), a combination of IGF-1 and BAPN, or untreated explants in medium containing 5% FBS [86]. De novo collagen synthesis and glycosaminoglycan (GAG) content were upregulated in IGF-1 treated explants although these effects were not significantly different in comparison with the control group cultured in media supplemented with AA and 5% FBS. Additionally, summed up mRNA signals from col I and col III in situ hybridizations showed increased detection in IGF-1 supplemented sections, but again, results were not significant, compared to untreated sections. Interestingly, addition of IGF-1 to BAPN treated cells was able to compensate the negative effect of BAPN on matrix synthesis to some extent. Although BAPN/IGF-1 treated cells did not reach GAG and collagen levels of untreated cells, the observed compensation effect supports an anabolic role in tendon metabolism. Histological analysis showed proliferation, margination and flattening of cells at the ends of control and IGF-1-treated explants to form endcaps. This flattening was more marked in the IGF-1 supplemented explants. Consistently, not only horse explants but also equine tenocytes cultured with 10, 50, or 200 ng/mL IGF-1 exhibited increased soluble collagen synthesis [82]. Effect was dose-dependent and the low dose of IGF-1 resulted in a significantly lower collagen synthesis than treatment with the medium and high dose. Experiments on equine tenocytes with 100 ng/mL IGF-1 confirm these findings, as collagen content as well as amount of GAGs were upregulated compared to untreated cells [76].

Supplementation with IGF-1 influenced also collagen deposition in *rat* tenocytes positively but in contrary to equine tenocytes, only low concentration of IGF-1 (10 ng/mL) significantly increased collagen deposition, whereas collagen content were not significantly higher with middle and higher dose of IGF-1 (50 and 100 ng/mL) [80]. On the contrary, addition of IGF-1 to canine tenocytes did not affect col I and col III synthesis. In Western Blot experiments with 3D high-density cultures of canine tenocytes treated with 10 ng/mL no collagen production could be detected [89]. On the other hand, Western Blot experiments with *mouse* tenocytes treated with 10 or 100 ng/mL IGF-1 did show procollagen Iα1 production after 24 h but the amount was not higher as in untreated tenocytes [12]. In short, many studies show a positive effect of IGF-1 on tenocytes and tendon fibroblasts regarding morphology, as measured collagen content was mostly upregulated in the performed experiments. This increase was often more prominent with time, weaker with higher FBS concentrations, missing in serum-free conditions and in experiments carried out with canine tenocytes. These different results pronounce the crucial role of the assessment regarding the outcome of the experiments.

#### 4.1.4. Differential Effects of IGF-1 Applied as a Single Factor to Different Cell Types—Association to Signalling Pathways

Before switching to combinations of growth factors with IGF-1 with the aim to stimulate tendon healing, we briefly sum up the differential effects of IGF-1 on tenocytes compared with MSCs and relate the findings to the corresponding signalling pathways. Among the signalling pathways triggering proliferation (PI3K/Akt/mTOR and PLC pathway, Figure 1), the comparison of MSCs with tenocytes revealed differential effects for different species, i.e., *rat*, *horse*, and human, respectively. In other words, depending from which species MSCs and tenocytes were harvested; in vitro treatment with IGF-1 (no other additional growth factors) had similar or different effects regarding cell proliferation.

As for cells derived from *horse*, the proliferation was enhanced by IGF-1 for both, equine bone marrow MSCs and tendon cells as well as tenocytes [76], implying similarity of these cell types with regard to activation of PI3K/Akt/mTOR and PLC pathways, respectively. Furthermore, when human ASCs were compared with human tenocytes, both cell types experienced an increase in proliferation, even both at best with the same IGF-1 concentration supplied [77]. This indicates a parallel and connated IGF-1 sensitivity for the associated pathways.

In contrast, the comparison of *rat* ASCs with *rat* tenocytes revealed only an increased proliferation for ASCs [79], but no effect on tenocytes—at any dose of IGF-1 [80]. Moreover, for *rat* tendon stem cells (TSCs), there was also no effect on proliferation [78] as found for the fully differentiated mature tenocytes. We conclude a species-dependency regarding differential effects when MSCs and tenocytes are compared regarding bioactivity of IGF-1 related to proliferation. Furthermore and specifically, for *rat* cells, only ASCs activate the PI3K/Akt/mTOR and PLC pathways upon IGF-1 binding, however, *rat* TSCs and tenocytes are not responsive in this regard. It has to be noted that although the PI3K/Akt/mTOR and PLC pathways are potentially involved in the activation of MSCs or tenocyte proliferation, it is not necessary that both pathways have to be activated for this induction; it may be that only one pathway is required. Further evaluation of relative importance could be performed with specific inhibitors for enzymes on each signaling pathway [22].

The synthesis of collagen as a result of IGF-1 binding can be associated to activation of PI3K/Akt/mTOR (Figure 1). Turning on collagen synthesis has been reported for equine tendon cells, however, not for equine bone marrow derived MSCs [76]. A direct comparison of these in vitro experiments is difficult, because for the tendon cells, the medium had been additionally supplemented with ascorbic acid (AA) besides IGF-1, and AA is a typical collagen booster [93]. Due to equal experimental conditions, a proper comparison is however enabled for the *rat* species. While *rat* tendon stem cells reacted towards IGF-1 with a downregulation of protein expression at 2 weeks, an upregulation was determined at 4 weeks, with 10 and 100 ng/mL, respectively [78]. For *rat* tenocytes on the other hand, there was an increase in collagen release already at day 3 [80]; however, only at a concentration of 10 ng/mL IGF-1. The *rat* tendon progenitors (TSCs) obviously needed more time to activate collagen synthesis as opposed to the fully differentiated tenocytes.

As for the gene expression of *COL1* and *COL3*, associated potentially with the Ras/MAPK and the PI3K/Akt/mTOR pathways, equine bone marrow MSCs and tendon cells, both supplemented with the same AA concentration besides IGF-1, did not show any effect (MSCs, 10_ng/mL IGF-1, day 10, and tenocytes, 100 ng/mL IGF-1, day 7) [76,88]. Nevertheless, collagen protein expression was enhanced in the experiment performed with equine tendon cells, implying that upregulation of gene expression was already over at these time points. Hence, dynamics seem to be a highly important parameter when different cell types are compared with respect to DNA replication.

Regarding cell migration associated with PI3K/Akt/mTOR and PLC pathways, in vitro experiments with cells stimulated by IGF-1 are rare. Equine tenocytes have been reported to better migrate into a GAG scaffold when they were stimulated by IGF-1 (dose-dependent) [82]; however, no further research has been performed regarding the effect of single factor application (IGF-1) on equine MSCs. Nevertheless, a study on IGF-1 induced migration of *rat* MSCs has reported an increase in cellular motility. The authors have reported that inhibition of the PI3K by LY294002 resulted in lower migration, giving evidence of activated PI3K/Akt/mTOR pathway [73].

Besides migration, IGF-1 is furthermore able to induce cell survival, particularly via the PI3K/Akt/mTOR pathway, where the phosphorylation of BAD suppresses apoptosis and in turn promotes cell survival (Figure 1). To compare effects of IGF-1 on different cell types, we were confronted with the fact that only human tenocytes were checked for cell survival upon IGF-1 administration–under serum-free conditions [84]. Interestingly, it has been reported that viability was maintained for 2 weeks, but was not accompanied by enhanced proliferation, which may be due to predominant Ras/MAPK pathway and to a lower degree to the BAD inhibition by the BCL-2 family–as otherwise Akt would activate proliferation of tenocytes parallel to supporting cell survival. To further examine relative contributions of Ras/MAPK and PI3K/Akt/mTOR pathways in cell survival, inhibitors such as PI3K inhibitor LY294002 or MAPK inhibitor PD98059 should be applied to these cell types, giving more profound molecular insight into the requirement and necessity of these pathways after IGF-1 supplementation [22].

### 4.2. IGF-1 in Combination with Other Growth Factors

Due to the anabolic effect of IGF-1 in tendon, big efforts have been made to further improve the influence of IGF-1. A widely used method is the combination of IGF-1 with other growth factors and synergistic effects could be shown in several studies.

#### 4.2.1. Proliferation and Migration

##### Stem Cells

**Figure 6:**
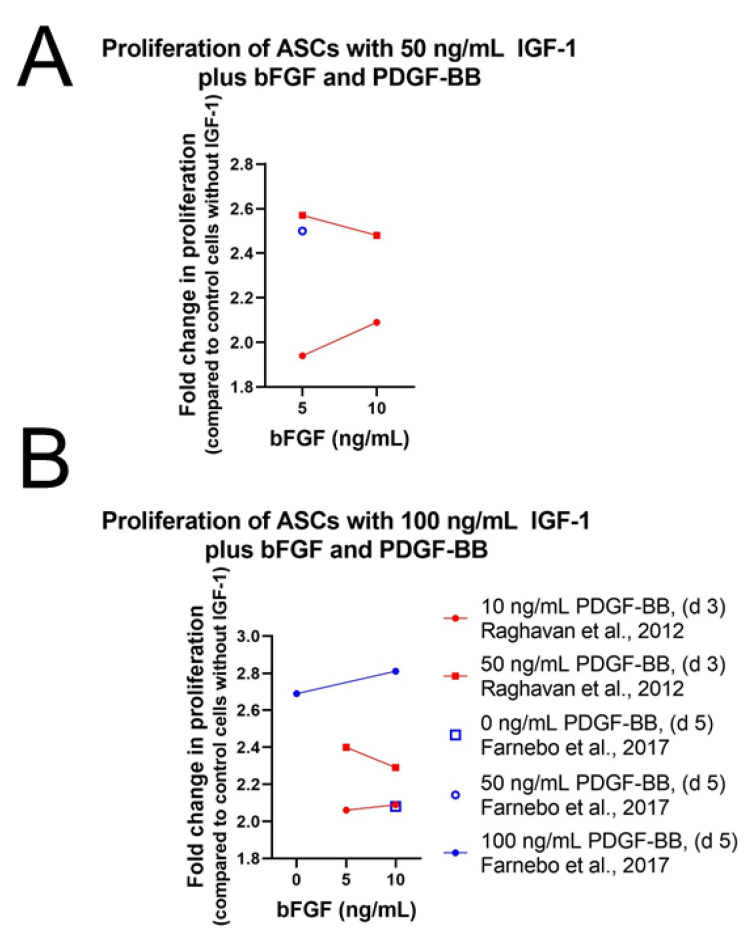
Impact of basic fibroblast growth factor (bFGF) concentration on the proliferation of ASCs in the presence of 50 ng/mL IGF-1 (**A**) or 100 ng/mL IGF-1 (**B**), respectively. Further information: Human ASCs in Ham’s F12 containing 0.2% BSA [77]; *rat* ASCs in ECM-solution containing 10% FCS [79].

A promising combination to enhance cell proliferation of stem cells is the use of IGF-1 together with PDGF-BB and bFGF (Table S3). Earlier studies have shown that PDGF-BB and bFGF are able to enhance tenocyte proliferation [93,94] and might therefore promote proliferation in MSCs. While IGF-1 supplementation alone had no effect on human ASCs, Raghavan et al. could show that the combination of IGF-1, PDGF-BB, and bFGF indeed increased cell proliferation in human ASCs [77]. However, not only was cell proliferation enhanced, but repopulation of tendon with ASCs was also significantly improved. The combination of 50 ng/mL IGF-1 with 50 ng/mL PDGF-BB and 5 ng/mL bFGF turned out to be the most effective combination for cell expansion and adhesion to a scaffold, two key steps in the reseeding of human tendon (Figure 6A). In *rat* ASCs treatment, 100 ng/mL IGF-1 was able to increase cell proliferation, but the combination of IGF-1 with PDGF-BB and bFGF was clearly more potent, confirming the results of Raghavan et al. [79] (Figure 6B). Although the combination of growth factors was the same as in human ASCs, the concentrations of the growth factors differed. In *rat* ASCs the most effective and efficient combination regarding proliferation was higher than in human ASCs and proved to be 100 ng/mL IGF-1, 10 ng/mL bFGF, and 100 ng/mL PDGF-BB, respectively.

Nevertheless, the combination of IGF-1 with PDGF-BB and bFGF proved to be a potent stimulant for ASC proliferation, while combination of IGF-1 (10 ng/mL) with bFGF (10 ng/mL) without PDGF-BB was not able to increase cell proliferation in equine MSCs [87]. In addition, low-level laser therapy (LLLT), a method able to improve proliferation and collagen synthesis in tenocytes [95], did not influence proliferation of equine MSCs positively.

##### Tenocytes and Fibroblasts

**Figure 7:**
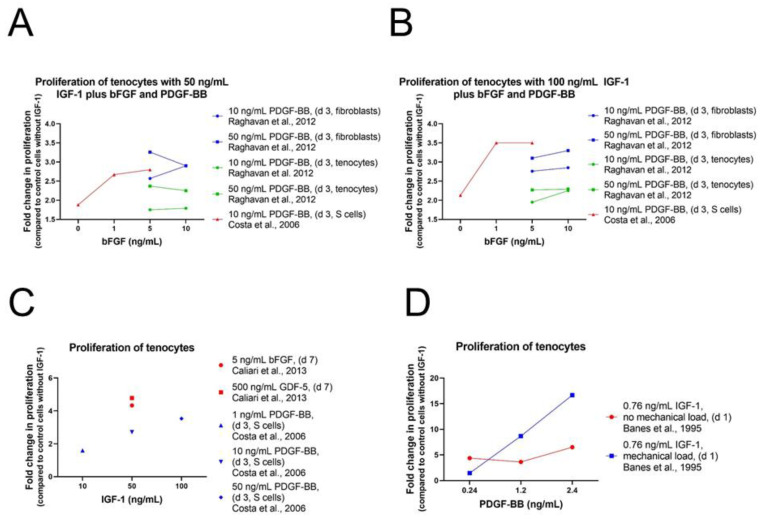
Impact of varying growth factor concentration on the proliferation of human tenocytes, cultivated in Ham’s F12 containing 0.2% BSA [77]; varying bFGF concentration in the presence of 50 ng/mL IGF-1 [81] (**A**) or 100 ng/mL IGF-1 [81] (**B**); varying IGF-1 concentration in the presence of several other growth factors [81,82] (**C**) and varying PDGF-BB concentrations in the presence of 0.76 ng/mL IGF-1 with and without mechanical loading (**D**) [13].

As in human ASCs, cell proliferation and repopulation of tendon scaffold were as well significantly increased in human fibroblasts and tenocytes using IGF-1 in combination with PDGF-BB and bFGF (Table S4). Consistently, 50 ng/mL IGF-1 with 50 ng/mL PDGF-BB and 5 ng/mL bFGF was the most potent combination for both effects [77] (Figure 7). Often only two growth factors are combined showing synergistic effects as well. Caliari et al. carried out experiments on equine tenocytes using IGF-1 (50 ng/mL) or PDGF-BB (50 ng/mL) in combination with bFGF (5 ng/mL) or together with growth differentiation factor 5 (GDF-5) (500 ng/mL), also known as BMP-14, an important signaling molecule in tenogenesis during development and in tendon healing after injury [96,97]. Cell number and metabolic activity were significantly higher in pairings with IGF-1 compared to non-supplemented media control, but levels were lower than in pairings with PDGF-BB instead of IGF-1 [82].

A synergistic effect could also be detected using the two proliferative factors IGF-1 and PDGF-BB on tenocytes from New Zealand white rabbits [81]. Already IGF-1 treatment showed a positive effect on cell proliferation, but combination of IGF-1 with PDGF-BB increased this effect clearly in a dose-response manner with highest proliferation levels using 100 ng/mL IGF-1 and 50 ng/mL PDGF-BB. Consistently with the results obtained in human ASCs, addition of bFGF (1 or 5 ng/mL) in combination with IGF-1 and PDGF-BB enhanced cell proliferation of rabbit ASCs further and showed highest levels using 100 ng/mL IGF-1 and 50 ng/mL PDGF-BB in combination with both concentrations of bFGF. Cells from the synovial sheath, epitenon, and endotenon were analyzed and although synergistic effect was observed among all three cell types, cell proliferation was most prominent for S-cells and had the weakest effect on T-cells (Figure 2B). The positive effect of IGF-1 in combination with PDGF-BB has already been reported earlier on avian cells from the epitenon and internal compartment (TSC) of flexor tendons [13]. A dose-dependent stimulation of DNA synthesis was observed using 100 pM (0.76 ng/mL) of IGF-1 in combination with 10, 50, or 100 pM (=0.24, 1.2, 2.4 ng/mL) PDGF-BB (Figure 7D). Despite the positive effect on cell proliferation levels were not as high as values in control cells cultured with serum. Cyclic mechanical load (0.05 strain; i.e., 5% maximum elongation, at 1 Hz for 8 h) stimulated DNA synthesis additionally in a synergistic fashion and values were higher for loads than without loads except for 10 pM PDGF-BB. Highest cell proliferation was reached using 100 pM IGF in combination with 100 pM PDGF-BB including mechanical loads. In addition, transforming growth factor beta 3 (TGFβ3) was used in combination with IGF-1 [84].

Members of the transforming growth factor β (TGFβ) family are involved in many cellular processes such as differentiation, extracellular matrix formation and the regulation of cell growth [98]. Although structures of the TGFβ members are very similar, some different effects could be detected. Amongst other things, it has been shown that TGFβ3 improves wound healing and reduces scar formation, while TGFβ1 and TGFβ2 are implicated in cutaneous scarring [99]. In contrast to the treatment with IGF-1 and PDGF-BB, no cell proliferation in human tenocytes could be observed upon IGF-1/TGFβ3 supplementation (10 ng/mL IGF-1 and 1 ng/mL TGFβ3 or 50 ng/mL IGF-1 and 10 ng/mL TGFβ3), but addition of IGF-1 together with TGFβ3 was capable to maintain tenocytes for 14 days in serum-free media. Consistently with IGF-1 treatment alone, cell number had significantly increased in culture media supplemented with 0% FBS plus IGF-1 and TGFβ3 compared with number in serum-free media and no growth factor supplementation where no cells were detectable. However, clearly highest cell number was achieved in the positive control cultures containing 10% FBS. Experiments were expanded in 3D culture using *bombyx* silk as scaffold. Results confirmed that differentiation in serum-free media containing 50 ng/mL IGF-1 and 10 ng/mL TGFβ3 was sufficient to allow human tendon regeneration in 2D and 3D conditions in the absence and presence of silk scaffold, respectively [100]. In summary, combinations of IGF-1 with PDGF-BB, bFGF, GDF-5, or TGFβ3 among others, show promising effects on tenocyte cultures with respect to proliferation under 2D and 3D conditions.

#### 4.2.2. Differentiation and Tendon Markers

##### Stem Cells

While supplementation with IGF-1 alone had only moderate effect on tendon markers in stem cells, combination of IGF-1 with other growth factor was more potent regarding expression levels of tendon related genes or proteins. One of these used growth factors is bone morphogenetic protein 12 (BMP-12), also known as growth differentiation factor 7 (GDF-7). Earlier studies have shown the capacity of BMP-12 to induce tenogenic differentiation of MSCs and to stimulate cell proliferation and collagen expression in human tendon fibroblasts indicating a critical role in tendon healing and regeneration [101,102].

**Figure 8:**
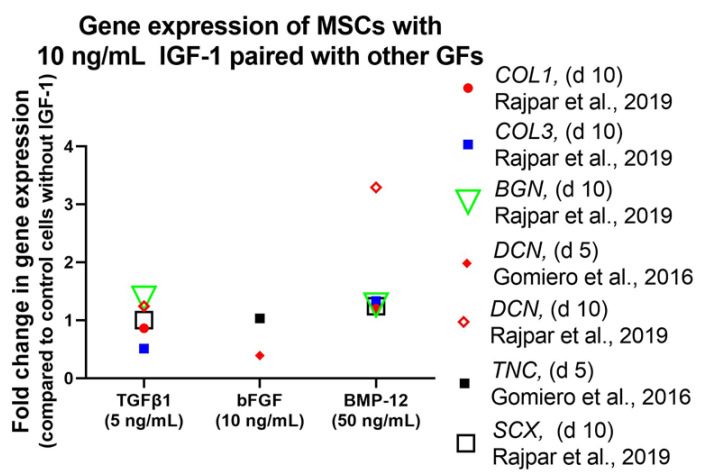
Impact of varying growth factor concentrations on the gene expression of human MSCs in the presence of 10 ng/mL IGF-1 and other growth factors as indicated. Further information: Equine MSCs in high-glucose DMEM containing 10% FBS and AA (37.5 µg/mL) [88] and equine tenocytes in DMEM containing 20% FCS [87].

Treatment of equine MSCs with the combination of 10 ng/mL IGF-1 and 50 ng/mL bone BMP-12 increased *DCN* gene expression significantly [88] (Figure 8). However, gene expression levels of *SCX*, *BGN*, *COL1*, and *COL3* remained unaffected. Furthermore, a second combination of IGF-1 with TGFβ1 (10 ng/mL and 5 ng/mL) did not increase gene expression of any analyzed marker. In addition to the combination with BMP-12 or TGFβ1, IGF-1 was also used together with bFGF. This combination (10 ng/mL each), was as well not capable to influence gene expression of *DCN*, *TNC*, and *EGR1* in equine MSCs positively and values stayed at the same level even in combination with LLLT [95]. While IGF-1 in combination with TGFβ1 showed no effect in equine MSCs on a genetic level, canine MSCs in 3D high-density cultures showed a positive effect upon IGF-1/TGFβ1 treatment (5 ng/mL each) on protein level [89]. Western Blot analysis showed highest DCN expression in tenocyte controls and MSCs treated with IGF-1/TGFβ1, where level was markedly increased after 14 days. TNMD expression was observed after 7 days, which increased to a value similar to control cultures at 14 days. In addition, SCX as well as col I and col III were upregulated in IGF-1/TGFβ1-treated MSCs and reached same intensities as tenocyte control cultures.

**Figure 9:**
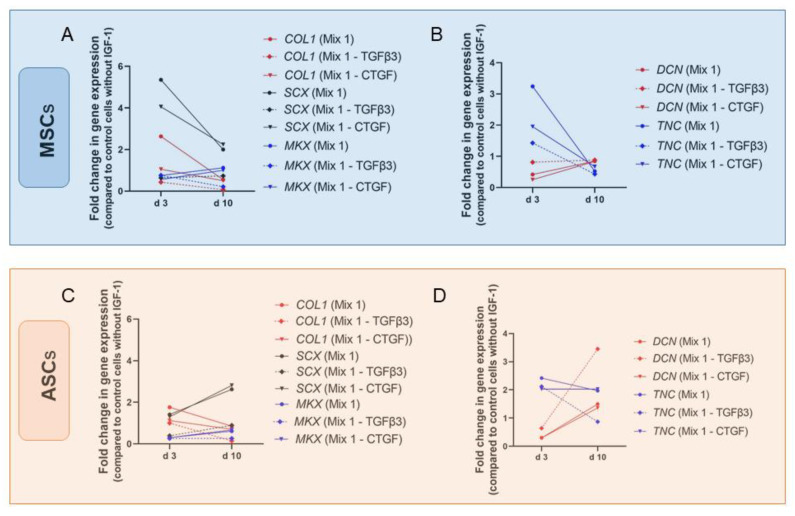
Impact of tenogenic media on the gene expression of human ASCs and MSCs. Gene expression of *COL1*, *SCX* and *MKX* for MSCs (**A**), *DNC* and *TNC* for MSCs (**B**), *COL1*, *SCX* and *MKX* for ASCs (**C**) and *DNC* and *TNC* for ASCs (**D**) are provided as manifold changes. Key: Mix 1 contains IGF-1 (50 ng/mL), BMP-12 (50 ng/mL), CTGF (100 ng/mL), bFGF (5 ng/mL), TGFβ3 (20 ng/mL), AA (25 µg/mL), and 1% FBS [103].

Importance of IGF-1 for tenogenic induction was also confirmed in human bone marrow-derived stromal cells (BMSCs) and ASCs. Perucca Orfei et al. tested several cell culture media containing different growth factors [103]. All mixtures contained 1% FBS, 25 µL/mL ascorbic acid (AA), 50 ng/mL PDGF-BB, 50 ng/mL BMP-12, and 5 ng/mL bFGF. Ascorbic acid plays an important role in wound healing and in collagen synthesis and therefore, AA is often present in tenogenic induction protocols [93,104,105]. Mixture 1 was additionally supplemented with 50 ng/mL IGF-1, 100 ng/mL CTGF, and 20 ng/mL TGFβ3. AA and CTGF are frequently used in differentiation media as it has been shown to activate tenogenic commitment and to improve tendon regeneration [106]. In mixture 3, TGFβ3 was missing and in mixture 5, CTGF was lacking otherwise they were identical to mixture 1. Mixtures without IGF-1 (mixture 4) and BMP-12 (mixture 2) showed no improvement on gene expression of *SCX* and *DCN* tenocytes and stem cells and therefore were not used for further experiments anymore. *SCX* expression was significantly increased in BMSCs after 3 days cultured with mixture 1 and 5 but not with mixture 3 lacking TGFβ3 though, this effect was not significant any more on day 10 (Figure 9). Levels of other markers such as *DCN*, Mohawk (*MKX*), *TNC*, and *COL1* were not significantly changed by the different treatments at any time point what was also the case for ASCs (Figure 9D). As in BMSCs, upregulation of *SCX* could be detected in ASCs with mixture 1 and 5. In contrast, increase was higher on day 10 than on day 3, and values were not significantly higher than in the control group.

Perucca Orfei et al. conclude that IGF-1 as well as BMP-12 and CTGF, emerged as being subordinate to TGFβ3 in the induction of tendon-specific transcription factors and may play an important role in the production of tendon-specific extracellular matrix. To summarize, IGF-1 in combination with other growth factors, such as BMP-12, PDGF-BB, bFGF, TGFβ1, and TGFβ3, can trigger tenogenic commitment of stem cells showing increased gene expression levels of typical tenogenic markers, such as *DCN* and *SCX*. However, results differ regarding growth factor combination, origin of cells, and duration of experiment.

##### Tenocytes and Fibroblasts

**Figure 10:**
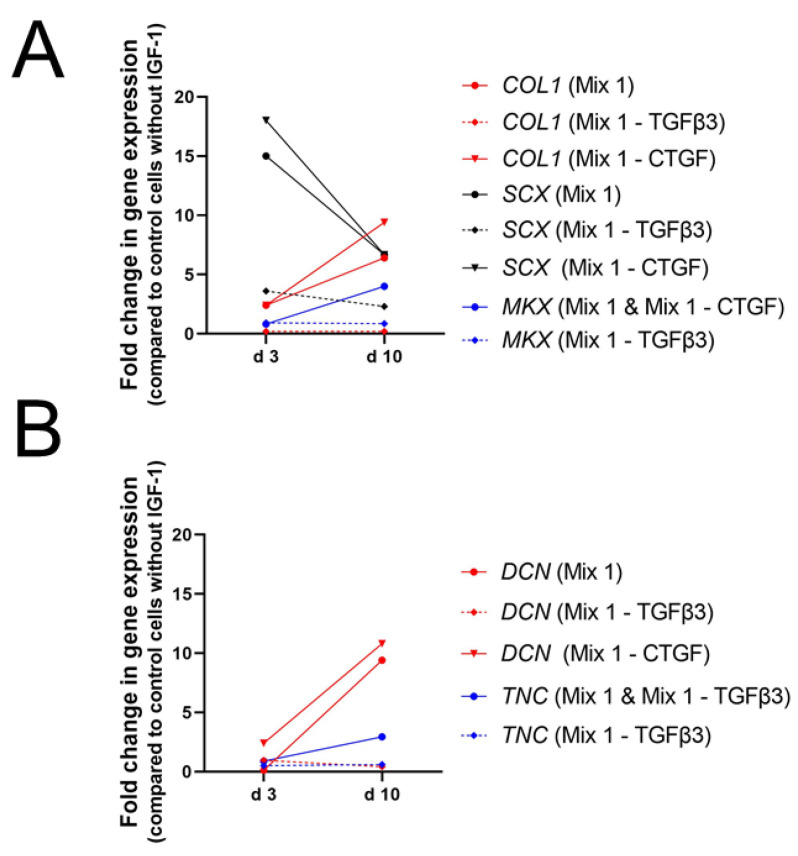
Impact of tenogenic induction media on the gene expression of human tenocytes. Gene expression of *COL1*, *SCX* and *MKX* (**A**) and *DNC* and *TNC* (**B**) are given in fold changes. Key: Mix 1 contains IGF-1 (50 ng/mL), BMP-12 (50 ng/mL), CTGF (100 ng/mL), bFGF (5 ng/mL), TGFβ3 (20 ng/mL), AA (25 µg/mL), and 1% FBS [103].

Growth factor mixtures used by Perucca Orfei et al. showed larger effect on human tenocytes than on BMSCs and ASCs. As in BMSCs, *SCX* expression was significantly increased after 3 days cultured with mixture 1 and 5 (Figure 10), but upregulation was two- to threefold higher than in BMSCs (Figure 9A). On day 10 however, effects were not significant. In contrast to stem cells, also gene expression level of *COL1* was significantly higher on day 3 with mixture 1 and 5 but not on day 10. On the other hand, *DCN* showed higher levels on day 10 than on day 3 with mixture 1 and 5 but upregulation was only significant compared to day 3 and not to control values. In contrast, *MKX* was significantly upregulated compared to control on day 10 with mixture 1.

Hence, the dynamics can be summed up as follows: Mixture 1, containing the complete set of growth factors used, increased gene expression of typical tendon markers like *SCX*, *COL1*, *DCN*, and *MKX*. However, effects turned out to be time-dependent and upregulation of *DCN* was not significant compared to control cells. Consistently, mixture 3, lacking TGFβ3, did not enhance expression levels of any marker [103].

**Figure 11:**
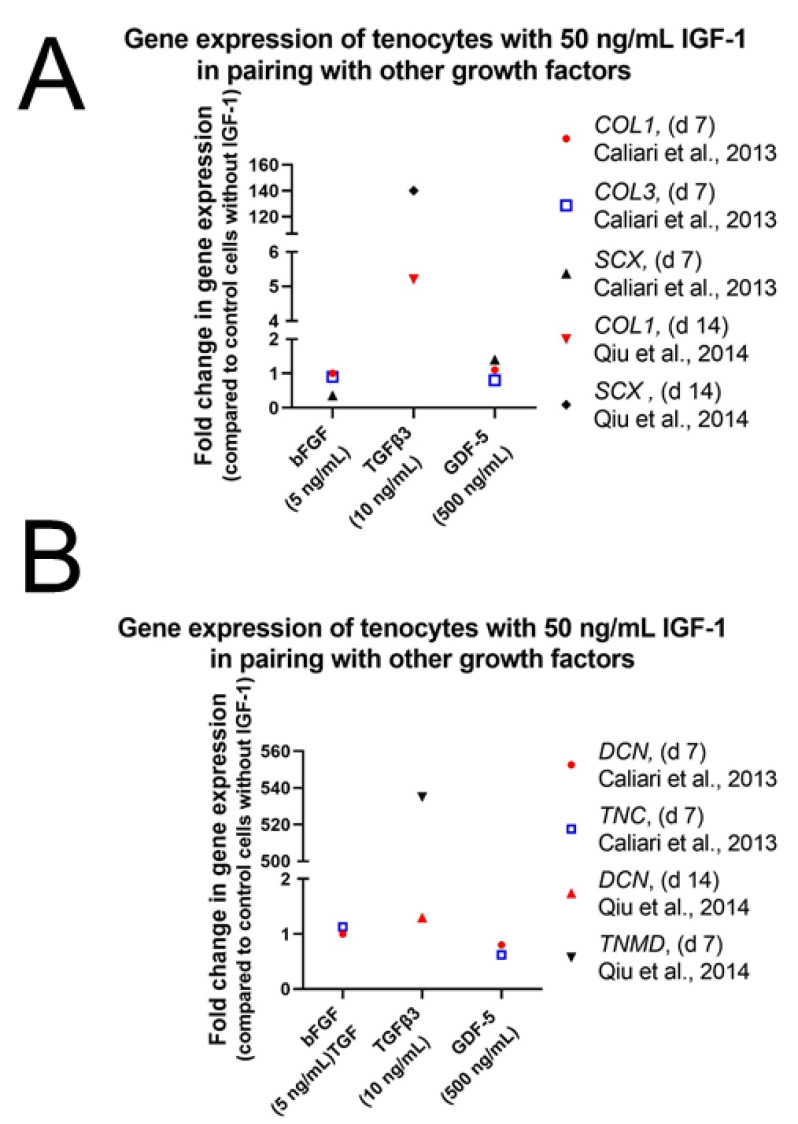
Impact of growth factor combinations on equine tenocytes in DMEM without FCS [82] and on human tenocytes in α-MEM without FBS [84]. Gene expression of *COL1* and *SCX* (**A**) and *DNC*, *TNC* and *TNMD* (**B**) are given in fold changes.

The important role of TGFβ3 in combination with IGF-1 has also been reported by Qiu et al. [84]. Human tenocytes in α-MEM without FBS but treated with 50 ng/mL IGF-1 and 10 ng/mL TGFβ3 increased gene expression levels of *SCX*, *COL1*, and *TNMD* remarkably after 14 days in culture (Figure 11), while supplementation with 50 ng/mL IGF-1 alone did not change expression levels of *SCX* and *COL1*. On the other hand, *DCN* was significantly increased upon treatment with 50 ng/mL IGF-1 and 10 ng/mL TGFβ3 but values were higher after supplementation with only 50 ng/mL IGF-1 (Figure 5D). Next to TGFβ3 also other growth factors were used with the goal to synergistically improve the effect of IGF-1. Whereas IGF-1 alone did not affect gene expression levels of tendon related genes in equine tenocytes, combination of 50 ng/mL IGF-1 with 500 ng/mL GDF-5 increased levels of *COMP* and *SCX* significantly (Figure 11A). Expression levels of *DCN*, *TNC*, *COL3* were not affected and though col I was slightly upregulated effect was not significant (Figure 11B). Pairings of IGF-1 (50 ng/mL) with bFGF (5 ng/mL) showed a weak but not significant increase of *TNC* but no influence on expression levels of all other markers could be detected [82].

We conclude from these studies that IGF-1 in combination with other growth factors plays an important role in tenogenic differentiation and especially GDF-5 and TGFβ3 seem to be potent candidates for this synergistic effect. Experiments, using mixtures supplemented additionally with PDGF-BB, BMP-12, bFGF, CTGF, and AA in combination with IGF-1 showed as well increased genetic expression levels of typical tendon markers such as *SCX*, *MKX*, and *COL1*.

#### 4.2.3. Morphology and ECM

##### Stem Cells

Canine MSCs treated with a combination of IGF-1 and TGFβ1 (5 ng/mL each) showed typical tenocyte like morphologies. Like tenocytes in the control culture, treated MSCs had a spindle shaped form with thin elongated processes, exhibited extensive intercellular contacts, and produced a well-organized extracellular matrix while untreated MSCs showed signs of apoptosis [89]. In addition, equine MSCs seeded on a collagen gel exhibited elongated cell morphology and no optical changes were observed regarding alignment between control MSCs and MSCs supplemented either with IGF-1/BMP-12 (10 ng/mL and 50 ng/mL) or IGF-1/TGFβ1 (10 ng/mL and 5 ng/mL). All groups uniformly integrated into the 3D gel constructs and progressively aligned with the longitudinal axis of tension. Gel contraction though, was highest on day 10 upon supplementation with IGF-1/TGFβ1 [88].

Furthermore, canine MSCs treated with IGF-1/TGFβ1 (5 ng/mL each) were capable to produce col I and col III comparable to control tenocytes, whereas no synthesis could be detected in untreated MSCs and MSCs treated only with IGF-1 [89]. Additionally, human ASCs repopulated on a tendon scaffold cultured with 50 ng/mL IGF-1, 5 ng/mL bFGF and 50 ng/mL PDGF-BB in Ham’s F12 media were able to synthesize collagen even after incubation for 12 days.

Immunohistochemical staining showed increased procollagen-1 content in treated cells compared to control cells but it is difficult to determine whether there is higher procollagen-1 production per cell [77]. Influence of IGF-1 could not only be detected on protein level, but also on gene expression levels of matrix related proteins. Rajpar et al. could show that treatment of equine MSCs with IGF-1/BMP-12 (10 ng/mL and 50 ng/mL) or IGF-1/TGFβ1 (10 ng/mL and 5 ng/mL) resulted in a twofold increase in GAG compared with control and single growth factor groups. Gene expression levels of col I and col III were upregulated after treatment with IGF-1/BMP-12 though not significantly compared to control.

On the other hand, supplementation with IGF-1/TGFβ1 did not affect expression of col I, while col III showed lower but not significantly reduced expression level [88]. These results demonstrate that IGF-1 in combination with TGFβ1 or BMP-12 is capable to direct morphology of stem cells towards a tendon like appearance going ahead with increased collagen production. Same effect could also be observed using IGF-1 in combination with PDGF-BB and bFGF.

##### Tenocytes and Fibroblasts

Typical tenocyte spindle-shape could also be maintained in human tenocytes in serum-free media supplemented with IGF-1 and TGFβ3 (10 ng/mL IGF-1 and 1 ng/mL TGFβ3 or 50 ng/mL IGF-1 and 10 ng/mL TGFβ3). After 14 day in culture tenocytes still exhibited elongated, spindle-shaped, fibroblast-like appearances and collagen staining showed thick, dense parallel collagen fibrils independent from the concentrations. The cell alignment and the collagen fibril morphology in these cultures were strikingly similar to those observed in the positive control with 10% FBS what could be confirmed by the quantitative analysis of the Sirius red staining. Collagen synthesis with the higher combination of IGF-1 and TGFβ3 was significantly higher than values with the lower concentration or in single growth factor groups. However, upregulation did not reach level of control tenocytes cultured with 10% FBS [84].

Procollagen synthesis has also been detected on tendon scaffold repopulated with human tenocytes and fibroblasts in media containing 50 ng/mL IGF-1, 5 ng/mL bFGF, and 50 ng/mL PDGF-BB [77]. Immunohistochemical staining of procollagen could not be analyzed quantitatively and therefore no statement could be made whether there is increased procollagen-1 production per cell when compared with the control. Soluble collagen content was as well measured in equine tenocytes with the combination of IGF-1 and bFGF (50 and 5 ng/mL) or IGF-1 with GDF-5 (50 and 500 ng/mL). Collagen synthesis was significantly increased for both soluble factor pairings compared to the control over the course of the 7-day experiment but the IGF-1/GDF-5 pairing induced a significantly greater amount of soluble collagen synthesis than pairing with IGF-1/bFGF, combinations with PDGF-BB instead of IGF-1 and non-supplemented control. However, on genetic level both IGF-1 supplemented groups showed no significant differences in expression of col I and col III compared with the control cells [82]. Overall, IGF-1 maintains tenocyte shape and increases collagen synthesis in combination with various growth factors. Convincing results were obtained using TGFβ3, GDF-5 and bFGF, the latter together with PDGF-BB, in combination with IGF-1.

### 4.3. Experiments Using Biological Supplementation Containing IGF-1

As reported, growth factors are widely used with the goal to improve tendon regeneration, but many of these are cost intensive recombinant proteins [107]. An alternative strategy in tendon healing is the use of cellular or acellular grafts, multipotent stem cells or plasma. Unfortunately, such experiments often differ in their preparation and application method and therefore results may differ as well.

PRP is an easily producible, low-cost source of several autologous growth factors [108]. Tenocytes treated with PRP showed increased cell proliferation and absolute col I synthesis was positively affected as well. On the other hand, relative col I synthesis normalized to cell proliferation was significantly reduced. Concentrations of measured growth factors were relatively low (IGF-1: 1–2 ng/mL, TGFβ1: 0.4–0.5 ng/mL, PDGF-AB: 0.2 ng/mL) but due to the factor combination, PRP turned out to be a potent stimulator for tenocytes [109].

In addition, human placental tissue grafts contain several factors that are potentially beneficial to tendon healing as indicated by recent studies [110]. McQuilling et al. could show that human tenocytes with hypothermically stored amniotic membrane (HSAM) or dehydrated amnion/chorion membrane (dACM) increased tenocyte proliferation and migration [111]. Like in PRP, concentrations of growth factors in the membranes measured were low (approximate concentrations of HSAM respectively dACM: IGF-1:3/12 ng/mL, bFGF: 1/2 ng/mL, bFGF: 0.02/0.04 ng/mL, PDGF-BB: 0/0.1 ng/mL, and TGFβ1: 1.2/3.2 ng/mL). Interestingly, levels of IGF-1 and bFGF were significantly lower in HSAM than in dACM leading to some different results. Tenocytes treated with dACM proliferated more robustly, whereas treatment with HSAM resulted in higher migration. Gene expression of col III, TNC and vascular endothelial growth factor (VEGF) was significantly increased with dACM, but significantly decreased with HSAM and consistently, soluble collagen content was increased only by dACM treatment. Both membranes resulted in altered tenocyte responses to inflammation with reduced TGFβ1 levels but dACM treatment resulted in increased expression and production of matrix metalloprotease-1 (MMP-1), whereas HSAM treatment decreased production of MMP-1.

Furthermore, co-culturing of tenocytes with adult multipotent stromal cells is increasingly used clinically to enhance tendon healing [112,113,114,115]. It has been shown that *horse* cells of the stromal vascular fraction (SVF) and adipose tissue-derived stromal cells (ASCs) express growth factors important in tendon healing such as IGF-1, TGFβ1, TGFβ3, FGF-2, and stromal cell-derived factor 1 (SDF-1) [116]. Conditioned media (CM) generated from ASCs showed significantly increased IGF-1 and SDF-1 concentrations on day 6 compared to day 3 and relative to the control medium on day 6. In addition, CM of high density seeded ASCs induced cell migration of ASCs in a dose-dependent manner. Co-culture of ASCs and tenocytes increased gene expression of col I and III, COMP, and DCN modestly in tenocytes. Co-culture of SVFs and tenocytes enhanced col I and III expression in tenocytes as well; however, this effect was only statistically significant for col III. On the other hand, COMP gene expression was decreased in tenocytes when co-cultured with SVFs at the highest seeding density. Although, results from co-culture experiments were less robust than expected, additional evidence of the potential anabolic influence of SVF and ASC could be provided. Obviously, big effort is made to improve tendon healing using biological supplementation and in the last years, achievements could be recorded.

However, it has to be emphasized that studies are complex and specific with regards to their conditions; due to the different experimental settings, concentrations of growth factors are very variable. In consequence, application of these methods is still limited in the presence and further investigations are needed.

## 5. PART B: Effects of IGF-1 Delivery In Vivo

For an analysis of the impact of IGF-1 it is not only important to understand the mechanism in vitro, but it is also mandatory to draw attention to in vivo experimentation. The in vivo studies examine the aspects what and how ongoing processes in the body may change and takes into account that the release kinetics of growth factors (GF) from tissue engineering vehicles can differ a lot compared to in vitro experiments. Under in vivo conditions, the GF is exposed to body fluids, enzymes, and other substances, which can alter the mechanism of action of the corresponding protein. Moreover, the sterilization method of the material has also an impact on degradation [117] and bioactivity of the incorporated GF [118,119].

Several in vivo studies where IGF-1 has been applied have shown its positive effect on tendon healing as the healing process was faster [14], the amount of DNA was higher, and tendons treated with IGF-1 exhibited better biomechanical properties [120,121,122]. However, as for the comparison of IGF-1 treated tissues with non-treated ones, it has been reported that histological sections exhibited only very small differences, which may be explained by the time point of the measurement; as most of the studies choose a rather late endpoint and therefore the tendon could already naturally heal to quite a good extent without IGF-1 application. In this regard, analysis at earlier time points may show a more differential result and hence a substantial difference between treatment and control.

### 5.1. IGF-1 Injections

In preclinical studies, a common application mode of IGF-1 to a tendon injury is by injection [123]. Some IGF-1 injections were accompanied by injections of other substances, prior [120] or during treatment of the wound site [14,122]. In addition, injections with growth hormone (GH) starting the release process of IGF-1 in the liver have been object of experimental studies [122,124].

In a study with a *rat* animal model, the plantaris tendon was excised in its entirety and 0.5 cm proximal to its insertion and the Achilles tendon was transected transversely [14]. Just before closing up completely 100 mL of inert 4% methylcellulose gel with 25 μg LR3-IGF-1 was injected (Table S5). The amount of IGF-1 was determined based on in vitro findings. The *rats* were analyzed functionally, biomechanically, histologically, and inflammatorily. The authors could show that the time until functional recovery was decreased by the additional injection of IGF-1. Fifty% functional recovery was achieved only on day 9 in the IGF-1 group, whereas in the transection group 50% functional recovery was achieved on day 13. There was also a trend to increased functional failure when a surgery was performed independent of the addition of IGF-1. For assessing the mode of action, an inflammatory agent (Carrageenan) was injected into additional rats. A functional deficit was achieved when injecting only Carrageenan, whereas with the addition of Carrageenan and IGF-1 this did not occur [14]. Carrageenan is a sulphated linear polysaccharide extracted from red seaweed and is known to induce an inflammatory response in laboratory animals [125]. After Carrageenan injection, the Achilles functional index returned to baseline already on day 2 for the IGF-1 group in contrast for the control group, where no return to baseline was observed during the period of 13 days. Further readouts, however, did not show any prominent effect of IGF-1 [14].

The injection of recombinant IGF-1 (rhIGF-1) was also tested in a *horse* model of flexor tendinitis [120]. This methodology included four injections every other day instead of one injection at the beginning as in the previously described *rat* model. Two μg rhIGF-1 divided into four injections were given to *horses* with collagen-induced flexor tendinitis. Results showed that the swelling was less in the IGF-1 treated group, and the lesions also, assessed by ultrasound, were smaller, especially after 4 weeks. Mechanical testing showed a trend towards stiffer tendons in the IGF-1 group. An important finding of this study was the increased DNA and hydroxyproline accumulation, which indicates a higher collagen content in the treatment group compared to the control that received no treatment. Moreover, the study has reported that IGF-1 displayed an anti-inflammatory effect when applied locally based on subjective observations of less edema and soft tissue swelling [120].

In another study on human patellar tendons, 40 human patients with diagnosed unilateral patellar tendinopathy were injected with 0.1 mL of 10 mg/mL rhIGF-1 or saline solution at the beginning of week 0, 1, and 2 [126]. The patients participated in a heavy slow resistance training after the first injection. After 12 weeks, a bilateral biopsy of the patellar tendons was analyzed. The *COL1A1* and *COL3A1* mRNA expression was significantly higher in the tendinopathic legs compared to the healthy legs. However, no difference in the ratio of *COL1A1* and *COL3A1* between tendinopathic and healthy leg was detected between IGF-1 treated and placebo group. By ultrasonographic analysis, the two treatment groups did not show a significant difference. The tendon thickness declined over time in the placebo group whereas, in the treatment group, the tendon remained the same size. The Victorian Institute of Sport Assessment—Patella (VISA-P) score was increased over time in the treatment and the placebo group, but at the 1-year follow up the VISA-P scores of the placebo group were higher, i.e., better outcome than the scores of the treatment group. The VISA-P is a questionnaire that assesses the symptoms, the ability to do sports and functionality during the study. Overall, there was no treatment benefit by the injection of rhIGF-1 on tendon healing [126].

### 5.2. GH Injections

Collagen is one of the most abundant proteins in the extracellular matrix of connective tissues, such as tendon tissue [1]. The increase of collagen synthesis and overall extracellular matrix synthesis after injury is an important step during healing. Especially in tendons where the healing capacity is rather low, it is important to have enough GFs to enhance the healing process by upregulated production of extracellular matrix. Natural tendons are composed mostly of col I whereas tendons after healing have also a high abundance of col III which has not the same properties as col I and may be part of a scar like tissue [127]. A solution could be the increase of col I synthesis in the early stage of healing with the aim to get a tendon with similar properties after healing as initially. In a placebo-controlled double-blinded study on human patellar tendon reported by Hansen et al. an increase in collagen synthesis after an injection of rhIGF-1 has been detected. Twelve healthy men aged 55 to 70 years received an injection 3 h and 24 h before the measurements. In one leg of the treatment group, 1 mg rhIGF-1 was injected into the patellar tendon, while the other leg received an equivalent amount of saline solution. The tendons were analyzed by stable isotope incorporation and microdialysis technique. Approximately 1–2 h after the last injection, the IGF-1 concentration in the interstitial tissue was increased in all the IGF-1 legs compared to the control legs and was also higher than the concentration of the circulating IGF-1. The tendon col I fractional synthesis rates were significantly higher in all the IGF-1 legs compared to the control legs 1.5 to 4.5 h after the last injection, but the difference between these two values was variable between different patients. The procollagen type I N-terminal propeptide which is an indirect marker for col I synthesis, was shown to be increased as well in the IGF-1 group [123].

Recent evidence suggests that the systemic administration of GH can enhance IGF-1, col I and col III mRNA expression. A study observed the effect of GH injections during 14 days on human patella tendon and quadriceps muscle. There was an increase of serum GH and IGF-1 for the treated group as well as an increase in expression of IGF-1. As the myofibrillar protein synthesis was not affected by the GH administration, Doessing and colleagues concluded that GH is especially important for strengthening the matrix tissue rather than the muscle tissue [124].

In another preclinical study, researchers have been working with GH, which was injected in order to induce the production of IGF-1 in vivo. The study was performed using a *rat* animal model and compared cut against not-cut Achilles tendons, with or without Botox injection. The injection of Botox ensured the complete unloading and therefore immobilization of the treated tendon. One half was treated with 2 mg GH/kg bodyweight daily, divided in 2 injections, while the other half remained untreated. After 10 days mechanical measurements were performed and a difference was determined between loaded and unloaded tendons. For the loaded group the stiffness decreased in GH treated groups compared to no GH whereas the other material properties were similar in both groups. The anabolic effect increased in the group with GH injection for all conditions confirmed by more rapid weight gain an increased weight of calf muscle, tibia, and femur. One of the most significant findings of this study was that loading has shown relatively more influence than application of GH on the healing capacity of the Achilles tendon [122].

### 5.3. Fibrin Sealing Gel

Besides the injection of GF a well-established method for GF is the incorporation of the GF into a fibrin gel which can then be applied at the wound site. In a study by Lyras et al., the effect of IGF-1 in combination with transforming growth factor β1 (TGFβ1) on mechanical properties and histology in rabbit patellar tendon was examined. Specifically, 4 ng of human recombinant TGF-β1 in combination with 25 μg of human recombinant IGF-1 were mixed into 2 mL of fibrin sealant, and fibrin gels with or without the GFs, respectively, were filled into the full thickness cut out defect of the patellar tendon. In the group with GFs, the ultimate stress, force at failure, energy uptake and stiffness increased significantly after 2 weeks. After 6 weeks however, there was no difference between treated and control patellar tendons. The evaluation of hematoxylin-and-eosin stained sections resulted in a higher number of newly formed vessels in the group with GF compared to the control group at two weeks, whereas at 6 weeks no difference has been observed [121]. These results suggest that IGF-1 and TGFβ1 in combination can enhance the early healing phase of tendons with increased, but transient angiogenesis and could therefore allow faster safe mobilization after injury, which has been reported to lead to increased and improved mechanical properties of tendons [128].

### 5.4. Platelet Rich Plasma

**Figure 12:**
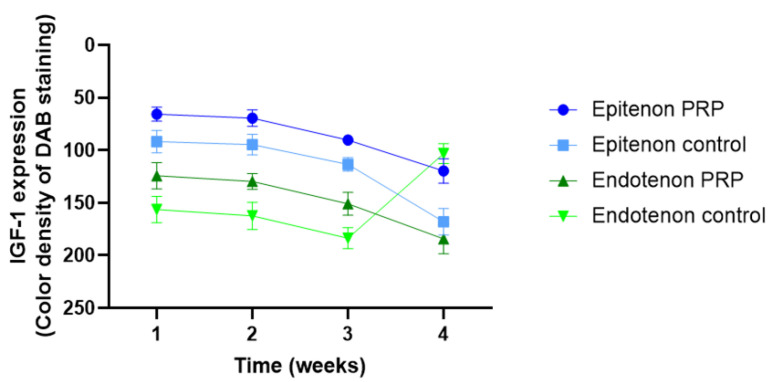
IGF-1 expression as a function of time in epitenon and endotenon for PRP treated specimen and control. Values are based on Table 1 in Reference [135]. High IGF-1 expression corresponds to low color density values. Lower color density values were attributed to higher brown intensity, whereas higher color density values corresponded to lower brown content.

A similar approach has been reported with PRP in a rabbit model. The PRP was obtained from each individual rabbit by extracting blood from the ear vein. In both legs, full thickness window defect in the mid part of patellar tendon was created, which was then filled with 2 mL of PRP gel and the overlying fascia was sutured in order to prevent outflow. The control group had the same surgical intervention but no PRP was added to the defect. The immunohistochemical IGF-1 expression was measured at 1, 2, 3, and 4 weeks after surgery and showed gradually lower IGF-1 expression in both groups for epitenon and endotenon (Figure 12). The expression of IGF-1 was generally higher in the epitenon than in the endotenon. In the first two weeks, the IGF-1 expressing cells were mainly inflammatory cells, endothelial cells, macrophages from granular tissue and irregular shaped tenocytes, whereas in the last 2 weeks IGF-1 expressing cells were mainly tenocytes. Due to metabolic shifts [129], wound macrophages have been reported to secrete various growth factors, such as PDGF, VEGF, and IGF-1 in order to mitigate hypoxia during injury and early healing phase [130]. It is noteworthy to mention that pro-inflammatory cytokines may dampen several components in the pathways downstream to IGF-1 binding [57]. Pro-inflammatory cytokines compete with IGF-1 intracellular signaling pathways by phosphorylating IRS (Figure 1) and thereby impeding the binding of IRS to IGFR1. On the other hand, macrophages that release IGF-1 counteract this cross-talk between pathways integral to growth/proliferation and the interfering pro-inflammatory cytokines. Upon binding of advanced glycation end-products known as AGEs [131] and increasingly present in an inflammatory milieu [132], macrophages release IGF-1 [133]. In addition, IGF-1 secretion has been described to induce T cell proliferation, B cell differentiation, and to slow down neutrophil apoptosis [134].

Coming back to the PRP study, it was interesting to note that at 4 weeks, IGF-1 was found to be less expressed in the endotenon with PRP compared to control, whereas in the epitenon it was the other way around. These results match with the reported suggestion that the epitenon cells start earlier with collagen production than endotenon cells [4]. The histological examination showed that the control group exhibited more immature tissue, whereas the PRP group tissue was denser, containing fewer less elastic fibers and more aligned tenocytes at week 3. At week 4, tendons treated with PRP were completely healed, whereas tendons of the control group lacked full recovery [135].

Next to PRP, a study with platelet rich fibrin (PRF) was performed [136]. The advantage of using PRF instead of PRP is the completely autologous self-assembly of PRF, whereas PRP in contrast still needs additional scaffolding due to its liquid character to prevent its diffusion to surrounding areas [137]. Initial in vitro experiments have shown a release of IGF-1 from the PRF in the first 48 h whereas other GFs, such as PDGF-BB, FGF-2, or TGFβ, were detectable for a period of at least six days. For the subsequent in vivo part, a 0.5 cm^3^ size defect was created in a rabbit Achilles tendon and filled up with PRF, heat denatured PRF (dePRF) or no filling (control). The study revealed that the repair zones of the PRF group contained mostly elongated tenocytes, the cellularity was higher than in the other two groups and the collagen fibers showed a crimping structure. On the contrary, the repair zone of the dePRF group was invaded with cells of different shape and size and fragmented collagen bundles. The overall histological score of PRF and dePRF were higher compared to control group. These findings could also be confirmed by the sonographic examination where the collagen fibers in the PRF group were well aligned and the defect area showed a continuous fibrillary structure [136].

### 5.5. Matrix Incorporation of IGF-1

A study focusing on the pegylation of IGF-1 applied a co-polymeric matrix based on poly(L-lactide)-co-poly(ε-caprolactone) with different modulations of incorporated IGF-1 to a rotator cuff defect in a *rat* model. Prabhath et al. incorporated the pulverized and lyophilized form of either pegylated IGF-1 mimic (PEG-IGF-1m), unpegylated IGF-1 mimic (IGF-1m), or recombinant human IGF-1 (rhIGF-1) to the matrix. The application of the PEG-IGF-1m matrix resulted in increased biomechanical properties of the tendon compared to repair control group and also could demonstrate with histological analysis the improvement of the healing after eight weeks. The collagen structure was denser and more organized in the PEG-IGF-1m group compared to all other groups, and the cellularity was decreased as well. The biomechanical testing reveled also improved results when rhIGF-1 was applied via the matrix but the extent of improvement was not as high as with the pegylated form of IGF-1. The researchers came to the conclusion that the usage of PEG-IGF-1m could be an effective approach for improvement of tendon healing because with the pegylation, the stability of IGF-1 can be improved and therefore a longer and more sustained release of the IGF-1 can be provided [138]. Besides IGF-1 application in rotator cuff repair, many other approaches have been recently reviewed [139].

## 6. Conclusions

To sum up the main information based on the literature research of IGF-1 used to improve tendon healing, it is obvious that this growth factor has been applied in an extensive amount of in vitro studies; however, in vivo studies have not that frequently been performed yet. While promising stimulating effects of IGF-1 have been shown for tenocytes of different species, its impact on mesenchymal stem cells is rather small and can only be improved by the combination of IGF-1 with other growth factors. Stimulating agents such as PRP have also been the focus of in vitro and in vivo research projects, but the variation in protocols to harvest PRP and the varying application conditions in the experiments do not allow drawing conclusions about its general (positive) impact.

From the studies we considered in this narrative review, we conclude that IGF-1 is a potent growth factor that can be used to stimulate tendon healing, particularly because of its anabolic and matrix stimulating effects. However, more preclinical in vivo studies have to be performed in order to pave IGF-1 application into the clinical setting.

## Data Availability

Not applicable.

## Supplementary Materials

The following supporting information can be downloaded at: https://www.mdpi.com/article/10.3390/ijms24032370/s1.

## Abbreviations

AA	ascorbic acid
AbAm	antibiotic antimycotic
ACAN	aggrecan
ALP	alkaline phosphatase
Akt = PKB	protein kinase B; collective name of a set of three serine/threonine-specific protein kinases
ASCs	adipose-derived stem cells, adipose tissue-derived stromal cells
α-MEM	α-MEM medium
BAD	cell death protein
BAPN	beta-aminopropionitrile
BCL-2	antagonist of cell death BAD
bFGF	basic fibroblast growth factor
BGN	biglycan
BMP	bone morphogenetic protein
BMP-12 = GDF-7	growth and differentiation factor-7
BMP-13 = GDF-6	growth and differentiation factor-6
BMP-14 = GDF-5	growth and differentiation factor-5
BMSC	bone marrow-derived stromal cells
BSA	bovine serum albumin
CG	collagen-glycosaminoglycan
CM	Conditioned media
col I	collagen I protein
col III	collagen III protein
*COL1A1*	collagen 1A1 gene
*COL3A1*	collagen 3A1 gene
COMP	cartilage oligomeric matrix protein
CSA	cross-sectional area
CTGF	connective tissue growth factor
dACM	dehydrated amnion/chorion membrane
DCN	Decorin
dePRF	denatured PRF = denatured platelet rich fibrin
DMEM	Dubelco’s modified Eagle Medium
DNA	desoxy ribonucleic acid
ECM	Extracellular matrix
EGF	epidermal growth factor
EGR 1	early growth response protein 1
FBS	fetal bovine serum
FCS	fetal calf serum
FGF	fibroblast growth factors
FOXO	forkhead box transcription factor
FSR	fractional synthesis rates
GAG	glycosaminoglycan
GDF	growth and differentiation factor
GH	growth hormone
Grb2	growth factor receptor-bound protein 2, an adapter protein
GTPase Ras	small GTPase (hydrolase enzymes for guanosine triphosphate)
Ham’s F12	Ham’s F12 cell culture medium
H&E	Hematoxylin&Eosin
HSAM	hypothermically stored amniotic membrane
HSR	heavy slow resistance
IGF	insulin-like growth factor
IGFBP	insulin-like growth factor binding protein
IGF-1Ea	insulin-like growth factor isoform Ea
IGF-1Eb	insulin-like growth factor isoform Eb
IGF-1Ec	insulin-like growth factor isoform Ec
IGF1R	insulin-like growth factor-1 receptor
IP3	inositol trisphosphate
IRS=	insulin receptor substrates
LLLT	low-level laser therapy
LR3-IGF-1	variant form of IGF-1 which binds with low affinity to circulating binding proteins
m	mimic
M-199	name of medium Mki67, proliferation Marker Ki67
MAPK	mitogen-activated protein kinase
MGF	mechano growth factor = insulin-like growth factor isoform Ec
Mki67	proliferation Marker Ki67
MKX	Mohawk
mRNA	messenger ribonucleic acid
MSC	mesenchymal stem cells
mTOR	mammalian target of Rapamycin
mTORC2	mammalian target of Rapamycin Complex 2
MTT	3-(4,5-dimethylthiazol-2-yl)-2,5-diphenyltetrazolium bromide…
OD	optical density
ON	Osteonectin
p85	regulatory subunit of PI3K
PDGF-BB	platelet-derived growth factor-BB
PDK1	Phosphoinositide-dependent Kinase-1
PEG-IGF-1m	pegylated IGF-1 mimic
PI3K	phosphatidylinositol-3 kinase
PINP	procollagen type I N-terminal propeptide
PIP2	phosphatidylinositol diphosphate
PIP3	phosphatidylinositol triphosphate
PLC	phospholipase C
PRF	platelet rich fibrin
PRP	platelet rich plasma
Raf	best characterized Ras effector
Ras	small GTPase
rhGH	recombinant human growth factors
rhIGF-1	recombinant human IGF-1
RTKs	receptor tyrosine kinases
RUNX	runt-related transcription factor
SCX	Scleraxis
SDF-1	stromal cell-derived factor 1
Sos	guanine nucleotide
SOX9	SRY-box transcription factor 9
SVF	stromal vascular fraction
TGFβ	transforming growth factor β
TGFβ1	transforming growth factor β1
TGFβ2	transforming growth factor β2
TGFβ3	transforming growth factor β3
TIF	tendon internal fibroblasts
TNC	Tenascin-C
TNMD	Tenomodulin
TSC	tendon-derived stem cells
VEGF	vascular endothelial growth factor
VISA-P	Victorian Institute of Sport Assessment-Patella
