# Supplementary material for: In Vitro and In Vivo Effects of IGF-1 Delivery Strategies on Tendon Healing: A Review
Source: Int J Mol Sci. 2023 Jan 25;24(3):2370. doi: 10.3390/ijms24032370 (PMC9916536; doi:10.3390/ijms24032370)
Supplement: Supplementary file 1 [file ijms-24-02370-s001.zip › ijms-2143710-supplementary.pdf]

International Journal of *Molecular Sciences*

MDPI

Supporting Information

Review

In vitro and *in vivo* effects of IGF-1 delivery strategies on tendon healing: a review

Iris Miescher [1,†], Julia Rieber [1,†], Maurizio Calcagni [1] and Johanna Buschmann [1,*]

Division of Plastic Surgery and Hand Surgery, University Hospital Zurich, Sternwartstrasse 14, 8091 Zurich, Switzerland

* Correspondence: johanna.buschmann@usz.ch; Tel.: +41-44-255-98-95
† Equal contribution

Tables S1 – S5

Table S1. Summary of *in vitro* tested conditions and cellular response of stem cells upon IGF-1 administration (sorted alphabetically according to cell species). Key: ↑ = increased, ↓ = decreased, → = no change , AA = Ascorbic acid, ASC = adipose-derived stem cells, BSA = bovine serum albumin, col = collagen, COMP = cartilage oligomeric matrix protein, DCN = Decorin, DMEM = Dubelco's Modified Eagle Medium, EGR1 = early growth response protein 1, FCS = fetal calf serum, Ham's F12 = Tenocyte culture medium, LLLT = low-level laser therapy, MSC = bone marrow-derived stem cells, ON = Osteonectin, SCX = scleraxis, TNC = tenascin-C, TNMD = Tenomodulin, TSC = tendon-derived stem cells.

***In vitro* model**	**IGF-1 [ng/mL]**	**Administration**	**Time point**	**Cellular response**	**Gene expression**
Canine MSCs [1]	10	3D high-density cultures on a nitrocellulose filter on a steel grid	7 d, 14 d	Similar morphology, intercellular contacts and extracellular organization like tenocytes<br>*Protein synthesis:*<br>col I *no*<br>col III *no*<br>DCN *no*<br>TNMD *no*<br>SCX *no*	
Equine bone marrow MSCs [2]	100	On tendon matrix, high-glucose DMEM with 10 % FBS	7 d	Proliferation ↑<br>Matrix proteins, col synthesis →<br>GAG synthesis ↑	*COL1*→<br>*COL3*→<br>*COMP* →
Equine bone marrow MSCs [3]	10	High-glucose DMEM with 10 % FCS and 37.5 µg/mL AA on a col I hydrogel	10 d	Matrix proteins:<br>GAG →<br>Gel contraction →	*COL1*→<br>*COL3*→<br>*DCN* →<br>*BGN* →<br>*SCX* →
Equine peripheral blood MSCs [4]	10	DMEM with 20 % FCS, + / - LLLT	5 d		*DCN* →<br>*TNC* →<br>*EGR1* →
Human ASCs [5]	10, 50, 100	Ham's F12 media with 0.2 % BSA	3 d	Proliferation ↑<br>(best with 50 ng/mL)	
Rat TSCs [6]	1, 10, 100	Basal tendon cell medium	14 d, 28 d	Proliferation → (any dose)<br>Maintenance of stem cell phenotype<br>↑ (d 28; 10 & 100 ng/mL)<br>*Protein expression* ***(same treatment*** *over time):*<br>col I ↓ (d 14);	

				↑ (d 28, 10 & 100)<br>col II ↓ (d 28),<br>→ (10 & 100)<br>SCX ↑ (d 28)<br>DCN ↑ (d 28, 1); →<br>TNC ↓ (d 28, 10); →<br>ON ↑ (d 14,);<br>↓ (d 14, 100); →<br>*(**same time point** compared to control):*<br>col I ↓ (d 14);<br>→ (d 28)<br>col II ↓ (d 14),<br>↑ (d 28, 10 & 100)<br>SCX ↑<br>DCN ↓ (d 14); ↑ (d 28)<br>TNC ↓ (d 28, 10); →<br>ON ↓ (d 14); ↑ (d 28)
Rat ASCs [7]	10, 50, 100	Reseeding in a tendon-specific hydrogel from human cadaver tendons in ECM solution with 10 % FCS	5 d	Proliferation ↑<br>(best with 100 ng/mL)

Table S2. Summary of *in vitro* tested conditions and cellular response of tenocytes and tendon fibroblasts upon IGF-1 administration (sorted alphabetically according to cell species). Key: ↑ = increased, ↓ = decreased, → = no change , α-MEM = α-MEM α-MEM Medium, AA = ascorbic acid, AbAm = antibiotic antimycotic, ACAN = aggrecan, ALP = alkaline phosphatase, BAPN = beta-aminopropionitrile, BSA = bovine serum albumin, col = collagen, COMP = cartilage oligomeric matrix protein, DCN = Decorin, dd = dose-dependent, DMEM = Dubelco's Modified Eagle Medium, GAG = glycosaminoglycan, FBS = fetal bovine serum, M-199 = name of medium Mki67 = marker of proliferation Ki-67, mRNA = messenger ribonucleic acid, SCX = scleraxis, SOX9 = SRY-box transcription factor 9, TNC = tenascin-C, TNMD = Tenomodulin, RUNX = runt-related transcription factor.

In vitro model	IGF-1 [ng/mL]	Administration	Time point	Cellular response	Gene expression
Avian tendon epitenon surface cells and tendon internal fibroblasts [8]	0.076, 0.38, 0.76	DMEM without FCS, mechanical load (1 Hz, 0.05 max. strain for 8 h)	1 d	Proliferation ↑ (higher with load than without; dd)	
Equine tendon cells [2]	100	On tendon matrix, high-glucose DMEM with 10 % FBS and 37.5 µg/mL AA	7 d	Proliferation ↑<br>*Matrix proteins :*<br>col synthesis ↑<br>GAG synthesis ↑	*COL1*→<br>*COL3*→<br>*COMP* →
Equine tenocytes [9]	10, 50, 200	Anisotropic GAG scaffolds, serum-free, non-supplemented DMEM	1 d (Chemotaxis)<br>7 d (Proliferation, gene expression)	Proliferation ↑ (dd)<br>Metabolic activity ↑ (dd)<br>Migration ↑ (dd)<br>Soluble col ↑	*COL1*↑<br>*COL3*↑<br>*SCX* ↓<br>*DCN* ↑ (low dose) / ↓<br>*TNC* ↑ (low dose) / ↓<br>*COMP* ↓
Equine explant culture from superficial digital flexor tendons [10]	250,<br>+/- 3 mg BAPN	Explant culture in M-199 media containing 100 µg/mL AA and 5 % FBS	10 d	Proliferation ↑<br>*Matrix proteins :*<br>col synthesis → ; GAG content →<br>In situ hybridization:<br>*col I* & *col III* mRNA →	
Human tenocytes [11]	10, 50	α-MEM with 0 % FBS<br>(control: 10 % FBS)	14 d	Survival of tenocytes without FBS for 14 days but no proliferation col layers less aggregated and weaker than control cells<br>Very low col synthesis<br>Spindle-shaped morphology	(50 ng/mL)<br>SCX →<br>COL1↑<br>TNMD →<br>*DCN* ↑

Human fibroblasts, and tenocytes [5]	10, 50, 100	Ham's F12 media with 0.2 % BSA	3 d	Proliferation ↑ (both cell types, best with 50 ng/mL)	
Human tenocytes [12]	2.5 x 10^{8}	DMEM / F12 with 0.5 or 10 % FBS in-coated plates with ~1.5 ml SYLGARD	7 d, 14 d, 10 d, 21 d, 28 d	Fibril diameter ↑<br>(d 21, not d 28)<br>Mean col content ↑	(0. 5% FCS, d 10, 14)<br>*COL1*↑<br>*COL3*↑<br>*SCX* ↑<br>*TNMD* ↑<br>*(10 % FCS, d 21, 28)*<br>*COL1* ↓<br>*COL3* ↓<br>*SCX* ↓<br>*TNMD* ↓
Mouse tenocytes [13]	100	Low-glucose DMEM with 1 % AbAm	1, 2, 6, 24 h	Proliferation ↑<br>(highest after 24 h)	(1h, 2h, 6h, 24 h)<br>*COL1A1*→<br>*COL1A2*→<br>*COL3*↑ (6 & 24 h *)*<br>*ki 67* ↑ (6 & 24 h)<br>*SCX* ↑ (1 & 2 h)<br>*TNMD* →<br>*BGN* ↓<br>*COMP* ↓
Rabbit tendon cells (synovial sheath (S), epitenon (E), endotenon (T)) [14]	10, 50, 100	Serum-free Ham's F12 with 0.2 % BSA	3 d	Proliferation ↑<br>(dd only synovial sheath cells)	
Rat tenocytes [15]	10, 50, 100	DMEM / F12 with 0.5 % FBS	3 d	Proliferation →<br>(at any dose)<br>col deposition ↑<br>(only with 10 ng/mL)	*COL1*→<br>*COL3*→<br>*DCN* →<br>*TNMD* ↑<br>*SCX* →<br>*RUNX* →<br>*ALP* ↑<br>*ACAN* ↑<br>*SOX9* ↓

Table S3. Summary of *in vitro* tested conditions and cellular responses on stem cells upon administration of IGF-1 in combination with other growth factors (sorted alphabetically according to cell species). Key: 🡑 = increased, 🡒 = no change, 🡓 = decreased, ,AA = ascorbic acid, ASC = adipose-derived stem cells, bFGF = basic fibroblast growth factor, BMP-12 = bone morphogenic protein-12 (= GDF-7), col = collagen, CTGF = connective tissue growth factor, BGN = Biglycan, BSA = bovine serum albumin, DCN = Decorin, ECM = extra cellular matrix, GAG = glycosaminoglycan, EGR1 = early growth response protein 1, FCS = fetal calf serum, LLLT = low-level laser technology, MSC = bone marrow-derived stem cells, PDGF-BB = platelet-derived growth factor, SCX = scleraxis, TGFβ = transforming growth factor beta, TNC = Tenascin-C, TNMD = Tenomodulin.

***In vitro* model**	**Growth factor [ng/mL]**	**Administration**	**Time point**	**Cellular response**	**Gene expression**
Canine MSCs [1]	IGF-1: 5<br>TGFβ1: 5	3D high-density cultures on a nitrocellulose filter on a steel grid	7 d, 14 d	Similar morphology, as tenocytes (spindle-shaped morphology, intercellular contacts, extracellular organization)<br>*Protein synthesis:*<br>col I yes<br>col III yes<br>DCN yes<br>TNMD yes<br>SCX yes	
Equine peripheral blood MSCs [4]	IGF-1: 10<br>bFGF: 10	DMEM with 20 % FCS, + / - LLLT	5 d	Proliferation 🡒	*DCN* 🡒<br>*TNC* 🡒<br>*EGR1* 🡒
Equine bone marrow MSCs [3]	IGF-1 & BMP-12: 10 & 50<br>IGF-1 & TGFβ1: 10 & 5	High-glucose DMEM with 10 % FCS and 37.5 µg/mL AA on a col I hydrogel	10 d	*Matrix protein:*<br>*GAG content* 🡑 *(both conditions)*<br>*Gel contraction* 🡑 *(IGF-1 & TGFβ1)*	a) **both conditions**<br>*BGN* 🡒<br>*SCX* 🡒<br>*COL1* 🡒<br>*COL3*🡒<br>*b)* ***IGF-1 & BMP-12***<br>*DCN* 🡑<br>*c)* ***IGF-1 & TGFβ1***<br>*DCN* 🡒
Human ASCs [5]	IGF-1: 50, 100<br>bFGF: 5, 10<br>PDGF-BB: 10, 50	Ham’s F12 media with 0.2 % BSA	3 d	Proliferation 🡑<br>(best with 50 ng/mL IGF-1, 5 ng/mL bFGF, and 50 ng/mL PDGF-BB)	
Human ASCs [5]	IGF-1: 50<br>bFGF: 5<br>PDGF-BB: 50	Repopulation on tendon scaffold in Ham’s F12	3 d	Proliferation 🡑 col production of reseeded cells continues on tendon scaffold after incubation of 12 days	

Human ASCs and MSCs [16]	IGF-1: 50<br>with BMP-12,<br>CTGF, TGFβ3,<br>AA, b-FGF	High-glucose<br>DMEM with 1 %<br>FCS	3 d, 10 d		*I*n IGF-1-free medium<br>(both cell types)<br>*SCX* →<br>*DCN* →
Rat ASCs [7]	IGF-1: 50, 100<br>bFGF: 5 10<br>PDGF-BB: 50, 100	Reseeding in a ten-<br>don-specific hydro-<br>gel from human ca-<br>daver tendons in<br>ECM solution with<br>10 % FCS	5 d	Proliferation ↑<br>(best with<br>100 ng/mL IGF-1,<br>10 ng/mL bFGF,<br>100 ng/mL PDGF-BB)	

Table S4. Summary of *in vitro* tested conditions and cellular responses on tenocytes and fibroblasts upon administration of IGF-1 in combination with other growth factors (sorted alphabetically according to cell species). Key: ↑ = increased, ↓ = decreased, → = no change, AA = ascorbic acid, bFGF = basic fibroblast growth factor, BMP-12 = bone morphogenic protein (= GDF-7), BSA = bovine serum albumin, col = collagen, COMP = cartilage oligomeric matrix protein, CTGF = connective tissue growth factor, DCN = Decorin, dd = dose-dependent, GAG = glycosaminoglycan, GDF-5 = growth differentiation factor, FBS = fetal bovine serum, FCS = fetal calf serum, PDGF-BB = platelet-derived growth factor, SCX = Scleraxis, TGFβ = transforming growth factor beta, TNC = tenascin-C, TNMD = Tenomodulin.

In vitro model	Growth factor [ng/mL]	Administration	Time point	Cellular response	Gene expression
Avian tendon epitenon surface cells and tendon internal fibroblasts [8]	IGF-1: 0.76<br>PDGF-BB: 0.24, 1.2, 2.4	DMEM without FCS, mechanical load (1 Hz, 0.05 max. strain for 8 h)	1 d	Proliferation ↑ (higher with load than without, PDGF-BB; dd)	
Equine tenocytes [9]	IGF-1 pairings:<br>IGF-1: 50<br>bFGF: 5<br>GDF-5: 500	Anisotropic GAG scaffolds, serum-free, non-supplemented DMEM	1 d (Chemotaxis)<br>7 d (Proliferation, gene expression	Proliferation ↑ (not as high as combinations of bFGF and GDF-5 with PDGF-BB)<br>Metabolic activity ↑ (not as high as combinations of bFGF and GDF-5 with PDGF-BB)<br>Soluble col ↑<br>(higher with GDF-5 than with bFGF)	a) **IGF-1 & bFGF**<br>*COL1* →<br>*COL3*→<br>*SCX* ↓<br>*DCN* →<br>*TNC* →<br>*COMP* ↓<br>b) **IGF-1 & GDF-5**<br>*COL1* →<br>*COL3*→<br>*SCX* ↑<br>*DCN* →<br>*TNC* →<br>*COMP* ↑
Human tenocytes [11]	IGF-1: 10, 50<br>TGFβ3: 1, 10	α-MEM with 0 % FBS<br>(control: 10 % FBS)	14 d	Survival of tenocytes without FBS for 14 d (no proliferation)<br>Spindle-shaped morphology<br>Cell alignment and col fibril morphology similar to control cells<br>col synthesis increased with combination of IGF-1 (50 ng/mL) and TGFβ3 (10 ng/mL) but still lower than control cells	Combination of IGF-1 (50 ng/mL) and TGFβ3 (10 ng/mL)<br>*SCX* ↑<br>*COL1* ↑<br>*TNMD* ↑<br>*DCN* ↑

Human fibroblasts and tenocytes [5]	IGF-1: 50, 100<br>bFGF: 5, 10<br>PDGF-BB: 10, 50	Ham’s F12 media with 0.2 % BSA	3 d	Both cell types:<br>Proliferation ↑<br>(best with 50 ng/mL IGF-1, 5 ng/mL bFGF, 50 ng/mL PDGF-BB)	
Human fibroblasts and tenocytes [5]	IGF-1: 50<br>bFGF: 5<br>PDGF-BB: 50	Repopulation on tendon scaffold in Ham’s F12	3 d	Both cell types:<br>Proliferation ↑<br>col production of reseeded cells continues on tendon scaffold after incubation of 12 d	
Human tendon cells [16]	IGF-1: 50<br>in different combinations with BMP-12, CTGF, TGFβ3, AA, bFGF	High-glucose DMEM with 1 % FCS	3 d, 10 d		In IGF-1-free medium:<br>*SCX* →<br>*DCN* →
Rabbit tendon cells (synovial sheath (S), epitenon (E), endotenon (T)) [14]	IGF-1: 10, 50, 100<br>PDGF-BB: 1, 10, 50<br>bFGF: 1, 5	Serum-free Ham’s F12 with 0.2 % BSA	3 d	Proliferation ↑ (best with 100 ng/mL IGF-1, 5 ng/mL bFGF, 50 ng/mL PDGF-BB)	

Table S5. Overview of *in vivo* experiments and outcomes after IGF-1 or GH administration, key: ↑ = increased, ↓ = decreased, → = no change, - = not assessed, col = collagen, DNA = desoxy ribonucleic acid, FSR = fractional synthesis rates, GH = growth hormone, H&E Hematoxylin&Eosin, HSR = heavy slow resistance, m = mimic, mRNA = messenger ribonucleic acid, PINP = procollagen type I N-terminal propeptide, PRF = platelet rich fibrin, PRP = platelet rich plasma, HSR = heavy slow resistance, rhGH = recombinant human growth factors VISA-P = Victorian Institute of Sport Assessment–Patella, GH = growth hormone, FSR = fractional synthesis rates, PINP = procollagen type I N-terminal propeptide, H&E Hematoxylin&Eosin . Outcomes noted in brackets are not statistically significant.

In vivo model	Growth factor concentration	Administra-tion	Time point	Biomechanics	Histology	Other outcomes
Rat Achilles tendon full transection [17]	25 µg LR3-IGF-1; 1 mg Carrageenan	Inert 4 % methyl-cellulose gel	15 d	*IGF-1*:<br>Time until functional recovery ↓<br>(Failure with surgery ↑)<br>Tendon length and circumference →<br>Failure loads →<br>Failure deformation →<br>Transection:<br>Gross stiffness ↓	*Transection*:<br>Altered tendon structure<br>Inflammatory reaction ↑	*IGF-1*:<br>Achilles index ↑<br>Functional deficit ↓<br>Neutrophilic invasion →
Rat Achilles ten-don full transection [18]	2 mg/kg bodyweight GH<br><br>1 U Botox/Muscle	Daily injection divided in 2 injections	10 d	*Loaded + GH + transection*:<br>Peak force, peak stress, elastic modulus, cross sectional area →<br>Stiffness ↓<br>*Loaded + GH + sham*:<br>Peak force, peak stress, elastic modulus, cross sectional area, stiffness →<br>*Unloaded + transection*:<br>Peak force, peak stress, stiffness elastic modulus ↓<br>Unloaded + sham:<br>Peak force, peak stress, stiffness elastic modulus →	-	*Loaded + GH*:<br>Anabolic effect<br><br>*Unloaded + GH*:<br>Anabolic effect

Rat rotator cuff tear [19]	rh-IGF-1, PEG-IGF-1m, IGF-1m	Matrix with incorporated IGF-1, sutured onto defect	4, 8 w	*PEG-IGF-1m*: Failure load, stiffness, work to failure, strength, Young's Modulus, toughness ↑	*Intervention*: Tissue healing → Cellularity ↑ remodeling ↑ *PEG-IGF-1m*: Col organized ↑ Round cell morphology ↑ Cellularity ↓ Col maturation ↑ *PEG-IGF-1m, IGF-1m*: Cellular orientation ↑	*PEG-IGF-1m*: Tendon typical appearance ↑ Cross sectional area ↓
Horse Flexor tendinitis [20]	2 µg rhIGF-1	1 injection divided into 4 small injections every second day for 10 times	0, 2, 3, 4, 6, 8 w	Load, load normalized by body weight, stress → IGF-1: (Stiffness ↑)	Cellularity ↑ *IGF-1*: Individual H&E scores for cell morphology, col fiber linearity, crimping, uniformity, density, inflammatory cells, neovascularisation, epitenon thickening → (Total H&E score ↑)	*IGF-1*: DNA and hydroxyproline ↑ GAGs → col I and col III → Limb circumference → Swelling ↓ Lesion size ↓
Rabbit Achilles tendon defect [21]	0.5 cm^3 PRF	Applied into defect	6 w	-	*PRF*: Repair zone filled with heterogeneous tissue, elongated cells High cellularity Dense collagen	*PRF*: Aligned tendon fibers Continuous fibrillary appearance

Rabbit Patellar Tendon Defect [22]	25 µg rhIGF-1<br>4 ng<br>TGF-β1	Fibrin sealant gel into the wound	2, 6 w	*IGF-1*:<br>Force at failure, ultimate stress, energy uptake, stiffness ↑ w 2<br>Force at failure, ultimate stress, energy uptake, stiffness → w 6	*IGF-1*:<br>Vessel number ↑<br>Plump shaped tenocytes ↑ w 2, → w 6<br>(Orientation of repair tissue ↑)	-
Rabbit Patellar Tendon Defect [23]	PRP enough to fill defect	Applied into defect	1, 2, 3 and 4 w	-	PRP:<br>Appearance of repair site → w 1-2<br>Denser tissue<br>Less elastic fibers ↓<br>Tenocyte orientation ↑<br>Immature tissue ↓ w 3<br>Completely healed w 4	*PRP*:<br>IGF-1 in epitenon & endotenon ↑ w 1-3<br>IGF-1 in endotenon ↓ w 4<br>IGF-1 in epitenon ↑ w 1-4<br>*Control*:<br>IGF-1 in epitenon ↑ w 1-3<br>IGF-1 in endotenon compared to epitenon ↑ w 4
Human patellar tendon [24]	1 mg rhIGF-1	Injection on day 1 and 2	d 2	-	-	*IGF-1*:<br>Interstitial tissue [IGF-1] locally ↑<br>Higher interstitial [IGF-1] than circulating [IGF-1]<br>Collagen FSR ↑ 1.5 – 4.5 h<br>PINP expression ↑ 2-3 h
Human patellar tendon [25]	0.1 mL of rhIGF-1 (10 mg/mL)	Injection at w 0, 1 and 2	12 w follow-up	-	-	*Tendinopathic*:<br>*COL1A1* & *COL3A1* ↑<br>(IGF-1Ea ↑)

	HSR training					*Total mRNA*<br>*IGF-1* ↑:<br>Doppler activity<br>↓ w 12<br>Tendon thickness →<br>Sports activity ↑<br>*Placebo*:<br>Tendon thickness ↓,<br>VISA-P ↑ w 0 and 1 y
Human patellar tendon [26]	33.3 µg/kg and<br>50 µg/kg bodyweight rhGH	Daily injection for 2 x 7 d, 5 months apart<br><br>d 1 - 7:<br>33.3 µg/kg<br>d 8 - 14:<br>50 µg/kg	d 14	-	-	*GH*:<br>Serum GH ↑<br>Serum IGF-1 ↑<br>Serum IGFBP-1 →<br>Serum IGFBP-3 ↑<br>*IGF-1Ea* mRNA↑<br>*IGF-1Ec* mRNA →<br>*COL1A1*mRNA and<br>*COL3A1* mRNA ↑<br>Tendon col protein FSR<br>↑

Figure S1

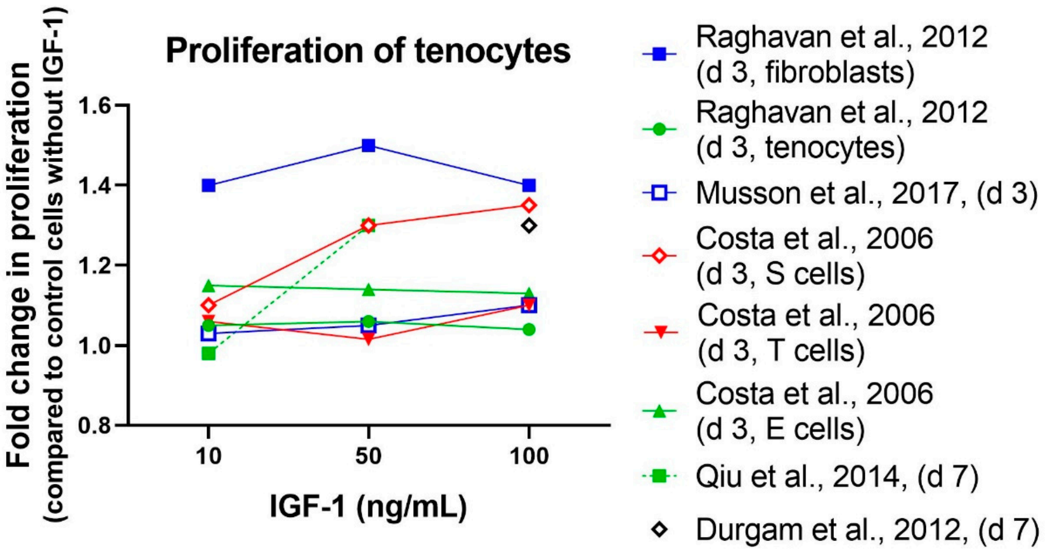

Figure S1: Proliferation of tenocytes as a function of IGF-1 concentration; smaller range of [IGF-1] compared to Figure 2B, in order to better distinguish the values between 10 and 100 ng/mL [2,5,11,14,15].

References cited in the table captions

1. Schneider, P.R., Buhrmann, C., Mobasheri, A., Matis, U., and Shakibaei, M., Three-dimensional high-density co-culture with primary tenocytes induces tenogenic differentiation in mesenchymal stem cells. *J Orthop Res*, **2011**, *29*(9), 1351-60.
2. Durgam, S.S., Stewart, A.A., Pondenis, H.C., Gutierrez-Nibeyro, S.M., Evans, R.B., and Stewart, M.C., Comparison of equine tendon- and bone marrow-derived cells cultured on tendon matrix with or without insulin-like growth factor-I supplementation. *Am J Vet Res*, **2012**, *73*(1), 153-61.
3. Rajpar, I. and Barrett, J.G., Optimizing growth factor induction of tenogenesis in three-dimensional culture of mesenchymal stem cells. *J Tissue Eng*, **2019**, *10*, 2041731419848776.
4. Gomiero, C., Bertolutti, G., Martinello, T., Van Bruaene, N., Broeckx, S.Y., Patruno, M., and Spaas, J.H., Tenogenic induction of equine mesenchymal stem cells by means of growth factors and low-level laser technology. *Vet Res Commun*, **2016**, *40*(1), 39-48.
5. Raghavan, S.S., Woon, C.Y.L., Kraus, A., Megerle, K., Hung, P., and Chang, J., Optimization of Human Tendon Tissue Engineering: Synergistic Effects of Growth Factors for Use in Tendon Scaffold Repopulation. *Plastic and Reconstructive Surgery*, **2012**, *129*(2), 479-489.
6. Holladay, C., Abbah, S.A., O'Dowd, C., Pandit, A., and Zeugolis, D.I., Preferential tendon stem cell response to growth factor supplementation. *J Tissue Eng Regen Med*, **2016**, *10*(9), 783-98.
7. Farnebo, S., Farnebo, L., Kim, M., Woon, C., Pham, H., and Chang, J., Optimized Repopulation of Tendon Hydrogel: Synergistic Effects of Growth Factor Combinations and Adipose-Derived Stem Cells. *Hand (New York, N.Y.)*, **2017**, *12*(1), 68-77.
8. Banes, A.J., Tsuzaki, M., Hu, P.Q., Brigman, B., Brown, T., Almekinders, L., Lawrence, W.T., and Fischer, T., PDGF-BB, IGF-I and mechanical load stimulate DNA synthesis in avian tendon fibroblasts in vitro. *Journal of Biomechanics*, **1995**, *28*(12), 1505-1513.
9. Caliari, S.R. and Harley, B.A.C., Composite Growth Factor Supplementation Strategies to Enhance Tenocyte Bioactivity in Aligned Collagen-GAG Scaffolds. *Tissue Engineering Part A*, **2013**, *19*(9-10), 1100-1112.
10. Dahlgren, L.A., Nixon, A.J., and Brower-Toland, B.D., Effects of beta-aminopropionitrile on equine tendon metabolism in vitro and on effects of insulin-like growth factor-I on matrix production by equine tenocytes. *Am J Vet Res*, **2001**, *62*(10), 1557-62.
11. Qiu, Y., Wang, X., Zhang, Y., Carr, A.J., Zhu, L., Xia, Z., and Sabokbar, A., Development of a refined tenocyte expansion culture technique for tendon tissue engineering. *Journal of Tissue Engineering and Regenerative Medicine*, **2014**, *8*(12), 955-962.
12. Herchenhan, A., Bayer, M.L., Eliasson, P., Magnusson, S.P., and Kjaer, M., Insulin-like growth factor I enhances collagen synthesis in engineered human tendon tissue. *Growth Horm IGF Res*, **2015**, *25*(1), 13-9.
13. Disser, N.P., Sugg, K.B., Talarek, J.R., Sarver, D.C., Rourke, B.J., and Mendias, C.L., Insulin-like growth factor 1 signaling in tenocytes is required for adult tendon growth. *Faseb j*, **2019**, *33*(11), 12680-12695.

14. Costa, M.A., Wu, C., Pham, B.V., Chong, A.K.S., Pham, H.M., and Chang, J., Tissue engineering of flexor tendons: Optimization of tenocyte proliferation using growth factor supplementation. *Tissue Engineering*, **2006**, *12*(7), 1937-1943.
15. Musson, D.S., Tay, M.L., Chhana, A., Pool, B., Coleman, B., Naot, D., and Cornish, J., Lactoferrin and parathyroid hormone are not harmful to primary tenocytes in vitro, but PDGF may be. *Muscles Ligaments Tendons J*, **2017**, *7*(2), 215-222.
16. Orfei, C.P., Vigano, M., Pearson, J.R., Colombini, A., De Luca, P., Ragni, E., Santos-Ruiz, L., and de Girolamo, L., In Vitro Induction of Tendon-Specific Markers in Tendon Cells, Adipose- and Bone Marrow-Derived Stem Cells is Dependent on TGF3, BMP-12 and Ascorbic Acid Stimulation. *International Journal of Molecular Sciences*, **2019**, *20*(1), 1-15.
17. Kurtz, C.A., Loebig, T.G., Anderson, D.D., DeMeo, P.J., and Campbell, P.G., Insulin-like growth factor I accelerates functional recovery from Achilles tendon injury in a rat model. *Am J Sports Med*, **1999**, *27*(3), 363-9.
18. Andersson, T., Eliasson, P., and Aspenberg, P., Growth hormone does not stimulate early healing in rat tendons. *Int J Sports Med*, **2012**, *33*(3), 240-3.
19. Prabhath, A., Vernekar, V.N., Esdaille, C.J., Eisenberg, E., Lebaschi, A., Badon, M., Seyedsalehi, A., Dzidotor, G., Tang, X., and Dyment, N., Pegylated insulin-like growth factor-1 biotherapeutic delivery promotes rotator cuff regeneration in a rat model. *Journal of Biomedical Materials Research Part A*, **2022**, *110*(7), 1356-1371.
20. Dahlgren, L.A., van der Meulen, M.C., Bertram, J.E., Starrak, G.S., and Nixon, A.J., Insulin-like growth factor-I improves cellular and molecular aspects of healing in a collagenase-induced model of flexor tendinitis. *J Orthop Res*, **2002**, *20*(5), 910-9.
21. Wong, C.C., Huang, Y.M., Chen, C.H., Lin, F.H., Yeh, Y.Y., and Bai, M.Y., Cytokine and Growth Factor Delivery from Implanted Platelet-Rich Fibrin Enhances Rabbit Achilles Tendon Healing. *Int J Mol Sci*, **2020**, *21*(9).
22. Lyras, D.N., Kazakos, K., Verettas, D., Chronopoulos, E., Folaranmi, S., and Agrogiannis, G., Effect of combined administration of Transforming Growth Factor-b1 and Insulin-like Growth Factor I on the mechanical properties of a patellar tendon defect model in rabbits. *Acta Orthopaedica Belgica*, **2010**, *76*(3), 380-386.
23. Lyras, D.N., Kazakos, K., Agrogiannis, G., Verettas, D., Kokka, A., Kiziridis, G., Chronopoulos, E., and Tryfonidis, M., Experimental study of tendon healing early phase: Is IGF-1 expression influenced by platelet rich plasma gel? *Orthopaedics & Traumatology: Surgery & Research*, **2010**, *96*(4), 381-387.
24. Hansen, M., Boesen, A., Holm, L., Flyvbjerg, A., Langberg, H., and Kjaer, M., Local administration of insulin-like growth factor-I (IGF-I) stimulates tendon collagen synthesis in humans. *Scandinavian Journal of Medicine & Science in Sports*, **2013**, *23*(5), 614-619.
25. Olesen, J.L., Hansen, M., Turtumoygard, I.F., Hoffner, R., Schjerling, P., Christensen, J., Mendias, C.L., Magnusson, P.S., and Kjaer, M., No Treatment Benefits of Local Administration of Insulin-like Growth Factor-1 in Addition to Heavy Slow Resistance Training in Tendinopathic Human Patellar Tendons: A Randomized, Double-Blind, Placebo-Controlled Trial With 1-Year Follow-up. *Am J Sports Med*, **2021**, *49*(9), 2361-2370.
26. Doessing, S., Heinemeier, K.M., Holm, L., Mackey, A.L., Schjerling, P., Rennie, M., Smith, K., Reitelseder, S., Kappelgaard, A.M., Rasmussen, M.H., et al., Growth hormone stimulates the collagen synthesis in human tendon and skeletal muscle without affecting myofibrillar protein synthesis. *J Physiol*, **2010**, *588*(Pt 2), 341-51.
